# Harnessing random peptide mixtures to combat multidrug-resistant fungal infections

**DOI:** 10.1128/mbio.00400-26

**Published:** 2026-04-14

**Authors:** John Adeoye, Yael Belo, Marina Campos Rocha, Hilla Hayby, Aygun Israyilova, Zvi Hayouka, Neta Shlezinger

**Affiliations:** 1 Koret School of Veterinary Medicine, Faculty of Agriculture, The Hebrew University 108750 , Rehovot, Israel; 2 Institute of Biochemistry, Food Science and Nutrition, The Robert H. Smith Faculty of Agricultural, Food & Environment, The Hebrew University of Jerusalem 274093, Rehovot, Israel; 3 Laboratory of Microbiology, Center for Excellence in Research, Development & Innovation, Baku State University 62384, Baku, Azerbaijan; Universidade de Sao Paulo Campus de Ribeirao Preto, Ribeirao Preto, Sao Paulo, Brazil

**Keywords:** antifungals, *Candida auris*, AMPs, candidiasis, AMR

## Abstract

Invasive fungal infections are associated with high mortality and are increasingly difficult to treat due to a limited antifungal arsenal and the rapid emergence of drug resistance. Novel therapeutic strategies that combine potent antifungal activity, low host toxicity, *in vivo* stability, and a reduced propensity for resistance development are urgently needed. Antimicrobial peptides (AMPs) stand out as a promising class of compounds to combat antimicrobial resistance. Leveraging the unique properties of AMPs, we previously developed a novel approach to synthesize random peptide mixtures (RPMs) with robust bactericidal activity against drug-resistant bacteria. Here, we evaluate the antifungal potential of RPMs and demonstrate species-dependent, broad-spectrum activity of FK20 (L-phenylalanine–L-lysine, 20-mer) against major human fungal pathogens, including *Candida* spp., *Cryptococcus neoformans*, and *Aspergillus fumigatus*, with particularly high potency against the multidrug-resistant pathogen *Candida auris*. Mechanistic analyses revealed rapid membrane and cell wall disruption accompanied by intracellular penetration, consistent with membrane-active antifungal activity. Importantly, experimental evolution assays demonstrated a markedly reduced capacity for resistance development in *C. auris*. FK20 inhibited biofilm formation and displayed substantial activity against mature, pre-formed biofilms, both alone and synergistically in combination with caspofungin. Finally, FK20 showed significant therapeutic efficacy in a murine model of systemic candidiasis. Collectively, these findings establish RPMs as a versatile antifungal platform with broad-spectrum activity, biofilm efficacy, and a low resistance footprint, highlighting their promise as a novel therapeutic strategy against drug-resistant fungal infections.

**IMPORTANCE:**

The rising prevalence of invasive fungal infections, particularly among immunocompromised individuals, has become a critical public health concern. However, antifungal drug development has not kept pace with this growing need, and treatment options remain limited to a small number of drug classes. The emergence of multidrug-resistant fungal pathogens, such as *Candida auris*, further exacerbates this crisis by reducing the efficacy of existing therapeutics and increasing the risk of treatment failure. In this study, we evaluate the antifungal potential of FK20, a random peptide mixture (RPM) composed of L-phenylalanine and L-lysine. FK20 displays potent activity against *C. auris* and other clinically relevant *Candida* species, impairs biofilm formation, and exhibits synergy with caspofungin. Importantly, FK20 limits the emergence of resistance and demonstrates therapeutic efficacy in a murine model of systemic candidiasis. These findings establish RPMs as a promising new class of antifungals with broad-spectrum activity and clinical potential against drug-resistant fungal infections.

## INTRODUCTION

The surge in antimicrobial resistance poses a significant threat to public health (1–4). While attention has predominantly centered on pan-resistant bacteria, mounting apprehension surrounds multidrug-resistant fungal pathogens, notably *Candida auris* (5). This emerging pathogen poses a significant global health threat due to its rapid worldwide spread and intrinsic multidrug resistance (6, 7). *C. auris* has been recognized as a serious global health threat by both national and international public health agencies (8, 9), underscoring the necessity for additional therapeutic options to address drug-resistant fungal infections.

*C. auris* was initially identified in 2009 in a Japanese hospital, where it was isolated from the external ear canal of a patient (10). Since then, genomic analysis has shed light on the concurrent emergence of distinct lineages, categorizing them into six major geographical clades across six continents over the past ~400 years (11–14). Its rapid transmission within clinical settings is attributed to its remarkable long-term persistence on surfaces, including human skin, as well as the widespread prevalence of drug-resistant isolates (15). Hospital-acquired *C. auris* infections, most commonly presenting as candidemia, occur predominantly in patients previously colonized with *C. auris*, most frequently on the skin, which serves as a persistent reservoir and a probable portal facilitating transmission and invasive disease, with reported mortality rates ranging from 30% to 60% (15, 16). Risk factors that increase susceptibility to *C. auris* infections are shared with other fungal infections and include iatrogenic, primary, or acquired immunodeficiency, prolonged antibiotic regimens, prolonged hospitalization, and invasive medical procedures (17).

*C. auris* is classified as a “superbug fungus,” posing an increasingly concerning threat to human health due to varying levels of intrinsic resistance. Approximately 5%–10% of isolates exhibit resistance to all three major antifungal drug classes commonly used in clinical settings (18, 19). It is evident that over 80% of *C. auris* isolates are resistant to at least one azole antifungal, most commonly fluconazole (20), and many strains exhibit notably elevated minimum inhibitory concentrations (MICs) for polyenes such as amphotericin B (21–23). For these reasons, echinocandins such as caspofungin are used as front-line therapeutics against systemic candidemia caused by *C. auris* (24–26). However, isolates with reduced susceptibility to one or more echinocandins have also been reported (14, 20, 25–28). Similar to other *Candida* species, *C. auris* employs diverse drug resistance mechanisms, including point mutations or chromosomal rearrangements affecting drug targets, as well as upregulation of efflux pumps. Collectively, the co-occurrence of multidrug resistance, rapid global emergence, and high mortality rates designates *C. auris* as a pathogen of significant clinical concern and complexity, highlighting the urgent need for novel antifungal strategies.

Current antifungal therapies are further limited by a narrow spectrum of drug classes, toxicity concerns, drug–drug interactions, and the emergence of resistance during prolonged treatment. In this context, alternative therapeutic paradigms that exploit fundamentally different modes of action are increasingly sought. One such strategy involves antimicrobial peptides (AMPs), an evolutionarily conserved component of innate immunity, including naturally occurring peptides such as defensins, cathelicidins, and histatins that are integral to innate immune systems across diverse organisms and exhibit broad-spectrum activity against bacteria, fungi, viruses, and parasites (29–32). AMPs are typically short amphipathic peptides rich in cationic and hydrophobic residues that exert antimicrobial activity primarily through interactions with microbial membranes, leading to membrane destabilization and cell death (33–35). Several endogenous and synthetic AMPs, including human defensins and antifungal proteins from filamentous fungi, have demonstrated antifungal activity against *Candida*, *Aspergillus*, and *Cryptococcus* species, underscoring the therapeutic promise of peptide-based antifungals (36–38).

Building on the fundamental principles underlying AMP activity, we previously developed a novel class of antimicrobial agents termed random peptide mixtures. RPMs were originally conceived as a synthetic alternative to natural antimicrobial peptides, aiming to overcome key limitations that have hindered AMP clinical translation, including high production costs and the rapid evolution of resistance associated with uniform, well-defined peptide sequences (39–41). RPMs are generated by solid-phase synthesis in which, at each coupling step, a defined mixture of one cationic and one hydrophobic amino acid is incorporated, resulting in a highly diverse ensemble of peptides with identical chain length, stereochemistry, and overall composition (42). This combinatorial design produces molecular heterogeneity that preserves antimicrobial potency while reducing selective pressure for resistance. RPMs have been shown to exhibit robust antimicrobial efficacy against both gram-positive and gram-negative bacteria *in vitro* and in multiple mouse infection models, including methicillin-resistant *Staphylococcus aureus* and *Acinetobacter baumannii* (43–46). Importantly, prior studies have demonstrated favorable safety profiles and limited host toxicity *in vivo*, supporting the translational potential of this approach.

Here, we present for the first time the antifungal activity of RPMs against the multidrug-resistant fungal pathogen *C. auris*. We demonstrate potent *in vitro* and *in vivo* efficacy of the RPMs, assess resistance development and collateral sensitivity, and explore synergistic interactions with established antifungals, thereby positioning RPMs as promising candidates for future antifungal development.

## RESULTS

### Random peptide mixtures demonstrate strong antifungal activity against *C. auris in vitro*

**Fig 1:**
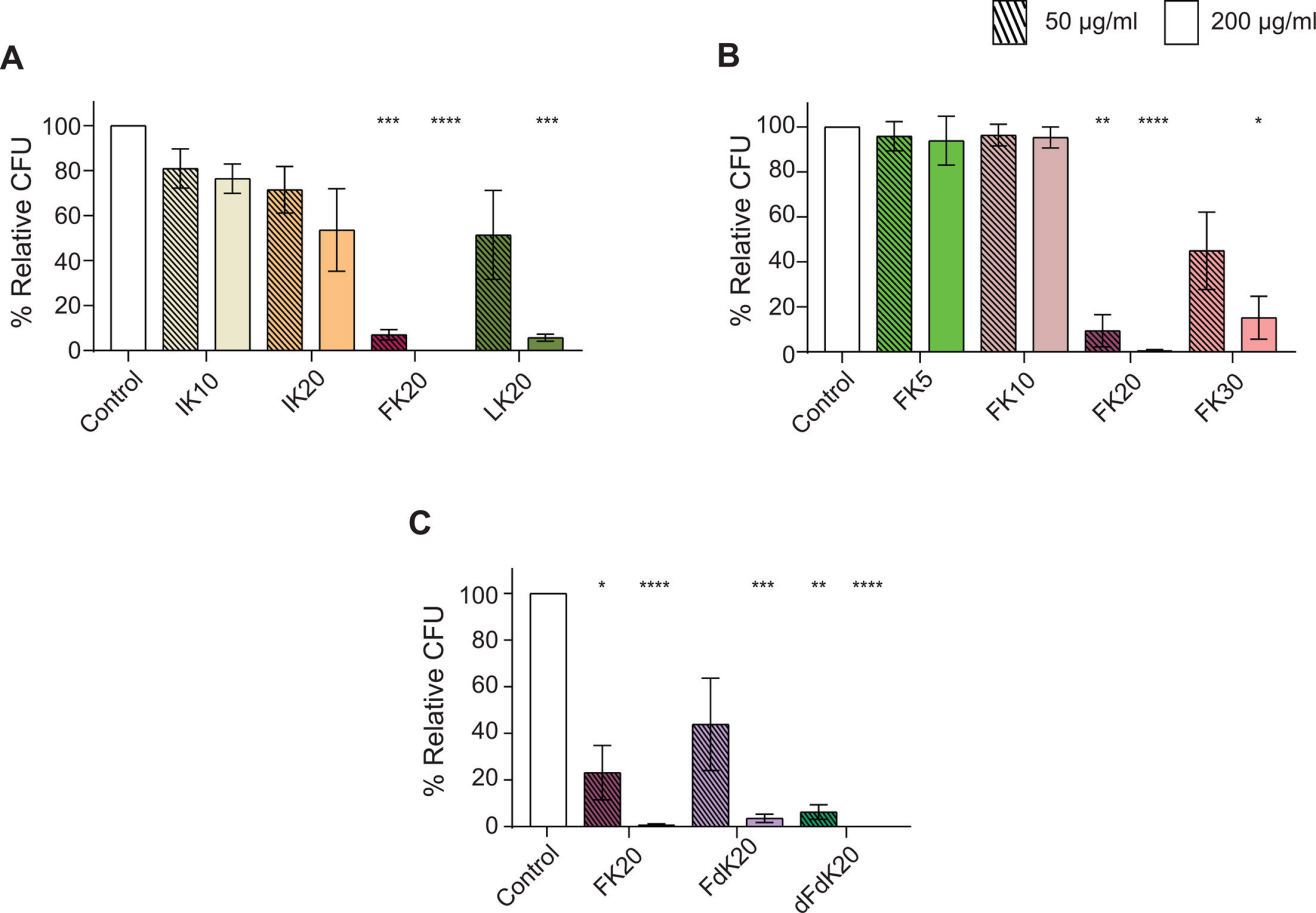
FK20 RPM exhibits potent antifungal activity against *C. auris. C. auris* cells (1 × 10^6^ cells/mL) were incubated with either 50 μg/mL (striped bars) or 200 μg/mL (solid bars) of the indicated RPMs for 45 min at 37°C with shaking (200 rpm) in PBS. Fungicidal activity was assessed based on (**A**) amino acid composition (IK10, IK20, FK20, and LK20), (**B**) peptide chain length (5-, 10-, 20-, and 30-mer FK), and (**C**) stereochemistry (FK20, FdK20, and dFdK20). Fungal viability was quantified by CFU enumeration and expressed as a percentage of untreated control. Data represent the mean ± SEM of three biologically independent experiments. Statistical significance was determined using Student’s *t*-test, comparing each treatment to the untreated control (set to 100% for each biological replicate). Significance levels are indicated as *P < 0.05, **P < 0.01, ***P < 0.001, and ****P < 0.0001; values without symbols are not significantly different from the control.

To elucidate the antifungal efficacy of random peptide mixtures against *C. auris*, killing assays were conducted using the clinical isolate B11117 to compare RPMs based on their amino acid composition, chain length, and stereochromy. Among the four tested RPMs composed of cationic and hydrophobic amino acids—FK20 (L-phenylalanine–L-lysine, 20-mer), LK20 (L-leucine–L-lysine, 20-mer), IK20 (L-isoleucine–L-lysine, 20-mer), and IK10 (L-isoleucine- L-lysine, 10-mer)—FK20 exhibited superior antifungal activity against *C. auris* (Fig. 1A). Given its minimal antifungal activity against *C. auris*, IK20 was selected as the negative control RPM. To further explore RPM optimization, the antifungal efficacy of FK RPMs with varying chain lengths was assessed (Fig. 1B). The 5- and 10-mer FK RPMs did not exhibit any discernible antifungal properties, whereas the 30-mer FK RPM showed marginal activity. In contrast, FK20 RPM demonstrated the highest antifungal activity, consistent with its previously reported broad antimicrobial potency and membrane-active properties (47). Next, the impact of FK RPM stereochemistry was assessed (Fig. 1C). Three stereoisomers were compared: FK20 (L-phenylalanine−L-lysine, 20-mer), FdK20 (L-phenylalanine−D-lysine, 20-mer), and dFdK20 (D-phenylalanine−D-lysine, 20-mer). The homochiral RPMs, FK20 and dFdK20, exhibited robust antifungal activity against *C. auris*, whereas the heterochiral RPM FdK20 displayed comparatively lower activity levels. Based on these findings, homochiral FK20 RPM was identified as the most effective candidate for combating *C. auris* and was selected for all subsequent experiments.

### FK20 RPM displays species-dependent antifungal activity across major fungal pathogens

**Fig 2:**
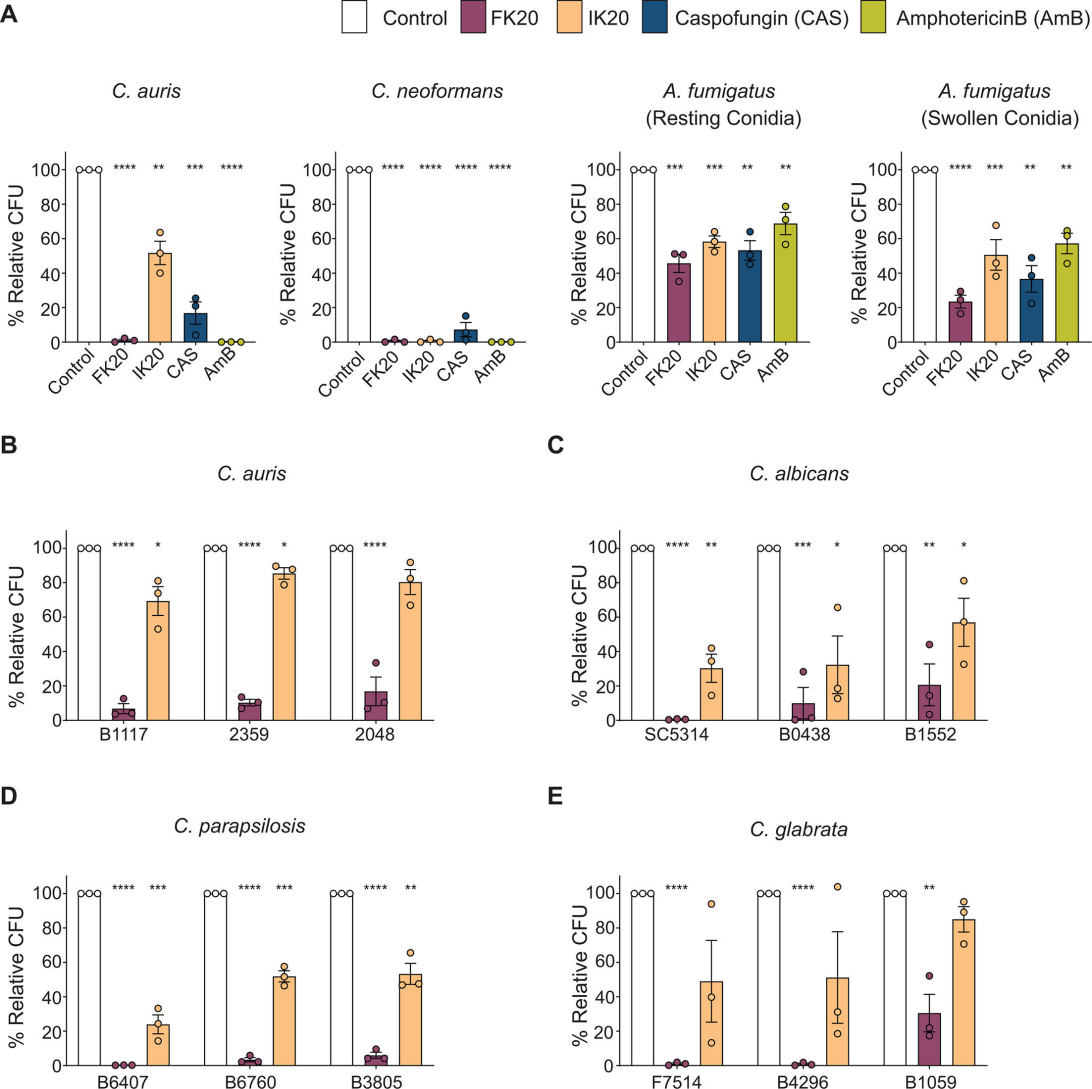
FK20 potency varies across major fungal pathogens of clinical relevance. (**A**) *C. auris* and *C. neoformans* yeast cells, as well as *A. fumigatus* conidia (resting and swollen), were incubated with 200 µg/mL FK20 or IK20 RPMs, 2 µg/mL caspofungin, 2 µg/mL amphotericin B, or PBS as a control for 5 h at 37°C, followed by CFU determination. (**B–E**) Cells of *C. auris* (**B**), *Candida albicans* (**C**), *Candida parapsilosis* (**D**), and *Candida glabrata* (**E**) were treated with 200 µg/mL FK20 or IK20 RPMs, or PBS as a control, for 45 min at 37°C, followed by CFU determination. Clinical isolates included *C. auris* skin-derived isolates (11117, 2359, and 2048); *C. albicans* blood-derived reference strain SC5314 and blood-derived isolates (B0438 and B1552); *C. parapsilosis* blood-derived isolates (B6407, B6760, and B3805); and *C. glabrata* isolates derived from peritoneal fluid (F7514) and blood (B4296 and B1059). Fungal viability is presented as CFUs expressed as a percentage of untreated control. Data represent the mean ± SEM of three biologically independent experiments. Statistical significance was determined using Student’s *t*-test, comparing each treatment to the untreated control (set to 100% for each biological replicate). Significance levels are indicated as *P < 0.05, **P < 0.01, ***P < 0.001, and ****P < 0.0001; values without symbols are not significantly different from the control.

To determine whether FK20 RPM activity extends beyond *Candida* spp., we evaluated its antifungal efficacy against additional clinically relevant fungal pathogens representing distinct phylogenetic and structural classes: *Cryptococcus neoformans* and *Aspergillus fumigatus*. FK20 RPM activity was compared to IK20 RPM and to the reference antifungals caspofungin and amphotericin B (Fig. 2A; Fig. S1A and B).

FK20 RPM exhibited potent fungicidal activity against *C. neoformans*, comparable to its activity against *C. auris*. In contrast, FK20 retained significant but reduced activity against *A. fumigatus*, achieving 77% killing of swollen conidia and 54% killing of resting conidia. IK20 RPM showed minimal fungicidal activity against *C. auris* and *A. fumigatus* (48% killing against *C. auris*, and 42% and 50% killing against resting and swollen *A. fumigatus* conidia, respectively), but retained high activity against *C. neoformans* (99% killing).

In comparison, standard antifungal agents displayed potent activity against the yeast pathogens *C. auris* and *C. neoformans*, but markedly reduced efficacy against the filamentous fungus *A. fumigatus* under the tested conditions. Amphotericin B exhibited strong activity against *C. auris* and *C. neoformans* (100% killing for both), but substantially weaker activity against *A. fumigatus* (31% and 43% killing against resting and swollen conidia, respectively). Caspofungin showed limited efficacy against *A. fumigatus* (47% and 63% killing against resting and swollen conidia, respectively) and moderate activity against *C. neoformans* and *C. auris* (93% and 83% killing, respectively), both lower than that observed for FK20.

Together, these data reveal marked species-specific differences in susceptibility to FK20 RPM, with the highest potency against *C. auris* and *C. neoformans* and reduced—yet clearly detectable—activity against *A. fumigatus*.

To further delineate FK20 RPM activity within the *Candida* genus, its antifungal efficacy was evaluated against clinical isolates of *C. auris* and other clinically relevant *Candida* species, including *Candida albicans*, *Candida parapsilosis*, and *Candida glabrata*. Cells were treated with 200 µg/mL FK20, IK20 RPM (control), or PBS. FK20 RPM demonstrated strong antifungal activity across all tested *Candida* species, with near-complete killing of all *C. parapsilosis* isolates and the majority of *C. auris*, *C. albicans*, and *C. glabrata* isolates (Fig. 2B through E). While the magnitude of killing varied between species and individual isolates, FK20 RPM consistently achieved substantial reductions in fungal viability across all tested clinical isolates. Together with its activity against non-*Candida* fungal pathogens, these findings establish FK20 RPM as a broad-spectrum antifungal agent whose efficacy is modulated by species- and isolate-specific properties.

### FK20 RPM disrupts *Candida auris* via cell wall penetration, membrane damage, ROS induction, and membrane depolarization

**Fig 3:**
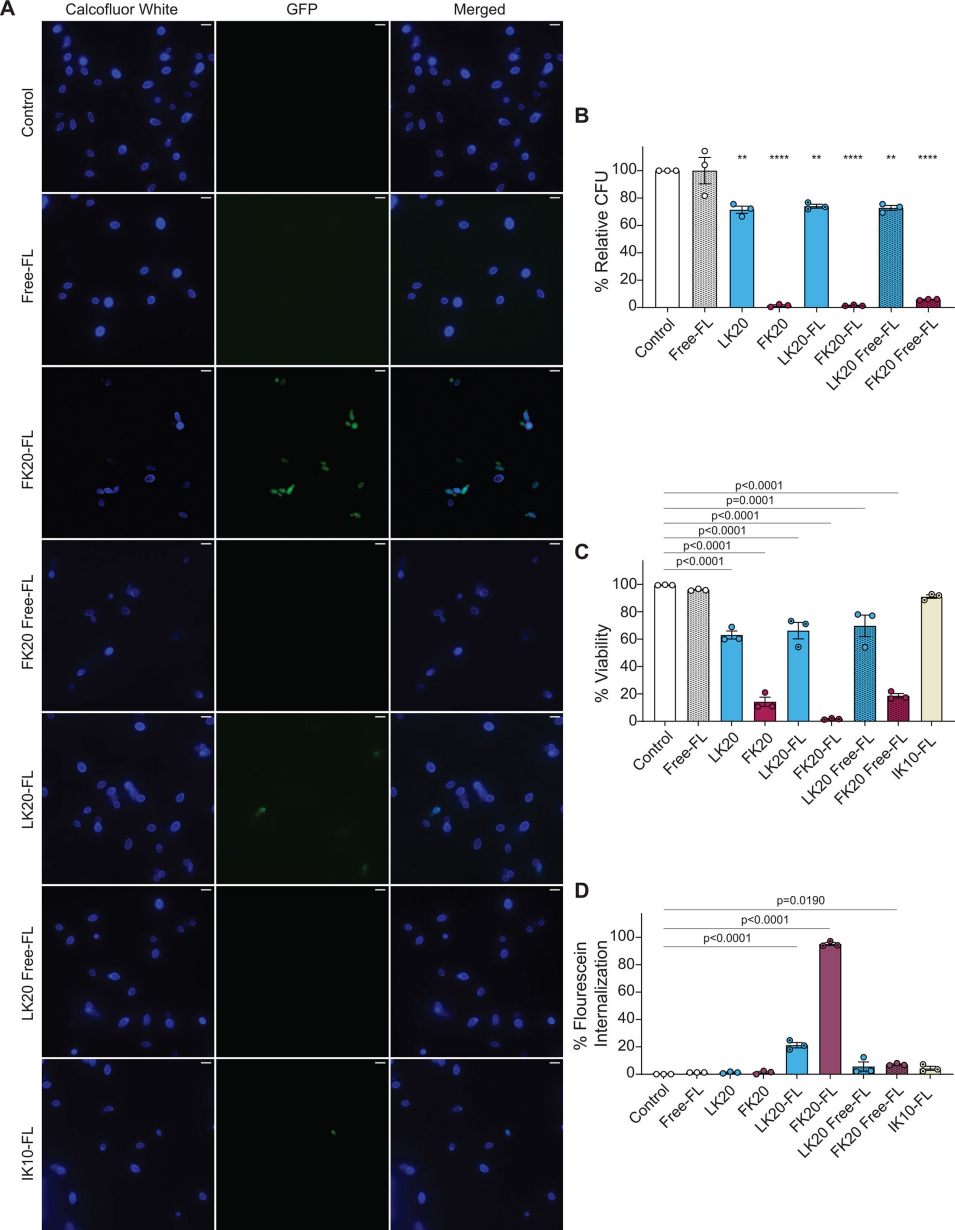
FK20 RPM penetrates *C. auris* cells. (**A**) *C. auris* cells (5 × 10^7^ cells/mL) were incubated with 200 µg/mL fluorescein-conjugated FK20, LK20, and IK10 RPMs for 45 min at 37°C in PBS. Controls included untreated cells, cells treated with free fluorescein alone, and cells treated with free fluorescein co-incubated with unlabeled FK20 or LK20. Cells were stained with Calcofluor White and visualized by fluorescence microscopy (100×, scale bar = 5 µm). Representative images from three independent experiments are shown. (**B and C**) Antifungal activity of fluorescein-labeled RPMs determined by (**B**) CFU enumeration and (**C**) PI staining. *C. auris* cells (1 × 10^6^ cells/mL) were treated as above; data represent mean CFU or percentage PI-negative (viable) cells ± SEM from three independent experiments performed in triplicate. (**D**) Internalization of fluorescein-conjugated RPMs assessed by flow cytometry. Data represent mean percentage ± SEM of FITC-positive cells from three independent experiments performed in triplicate. Statistical significance was determined using Student’s *t*-test, comparing each treatment to the untreated control (set to 100% for each biological replicate). Significance levels are indicated as *P < 0.05, **P < 0.01, ***P < 0.001, and ****P < 0.0001; values without symbols are not significantly different from the control.

**Fig 4:**
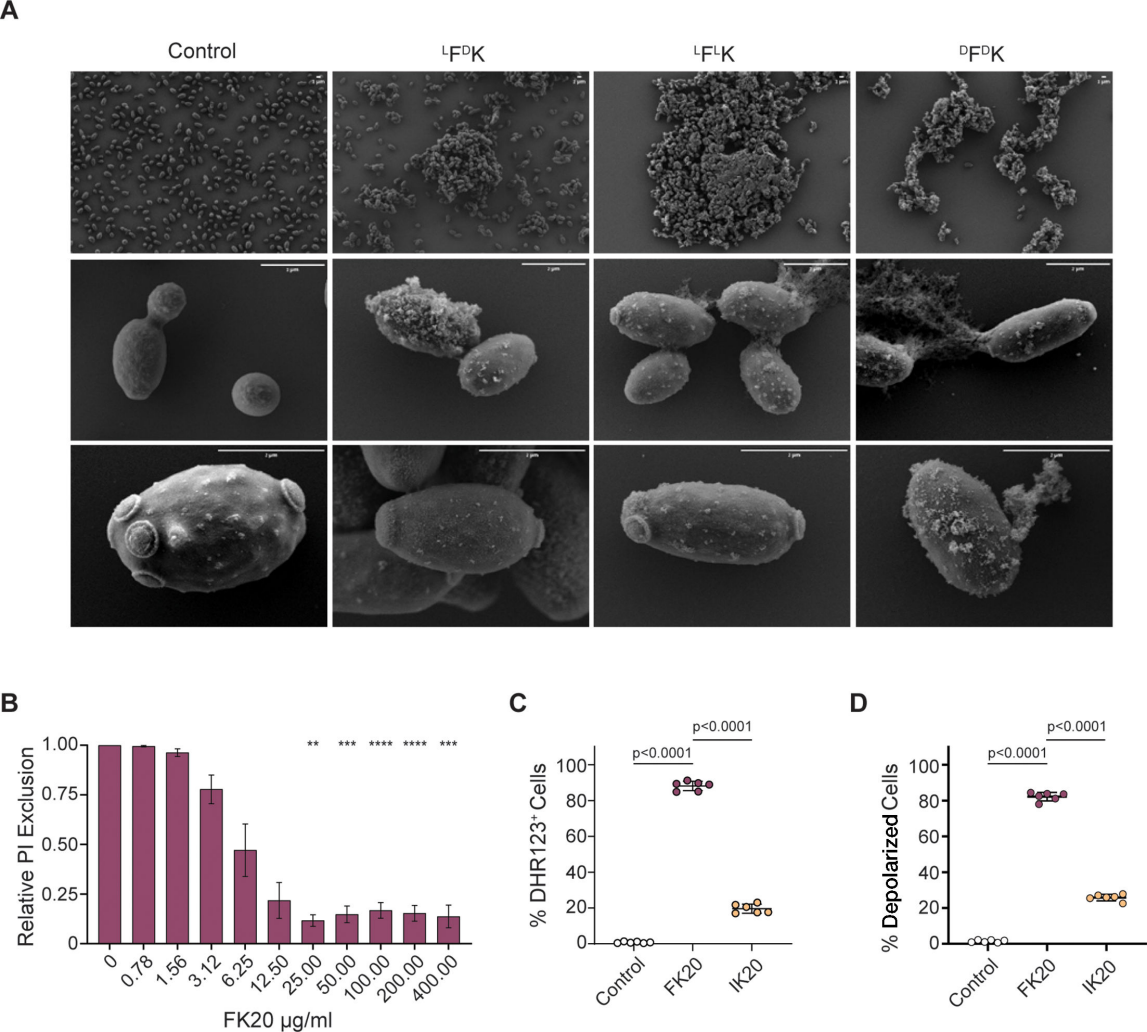
*C. auris* cell surface after interaction with 20-mer FK RPMs. (**A**) Scanning electron microscopy of untreated *C. auris* and cells treated with 200 μg/mL of 20-mer FK20, FdK, or dFdK using JEOL JSM-7800f scanning electron microscope at 20 kV (scale bar = 1 µm). Representative micrographs from three independent experiments are shown. (**B–D**) FK20 induces membrane damage, reactive oxygen species (ROS) generation, and membrane depolarization in *C. auris* cells. Cells (1 × 10^6^ cells/mL) were treated with FK20 for 45 min at 37°C in PBS. Cells were analyzed using flow cytometry. (**B**) Dose-dependent membrane damage induced was determined by incubating cells with FK20 at concentrations ranging from 0 to 400 µg/mL. Membrane integrity was assessed by PI uptake into the cells (flow cytometry). Data are presented as PI-excluded cells (PI negative) relative to untreated control. (**C**) Intracellular ROS generation was assessed by staining *C. auris* cells with DHR 123 after treatment with FK20. (**D**) Membrane depolarization was assessed using DIBAC_4_(5) following FK20 treatment. Data represent mean ± SEM from three independent experiments performed in triplicate. Statistical significance was determined by one-way ANOVA with Tukey’s multiple comparisons test. Significance levels are indicated as *P < 0.05, **P < 0.01, ***P < 0.001, and ****P < 0.0001; values without symbols are not significantly different from the control.

To elucidate the mechanisms underlying FK20 RPM’s antifungal activity, its interaction with *C. auris* cells was examined using a combination of fluorescence microscopy, flow cytometry, and membrane integrity assays. Because RPMs are designed to mimic the physicochemical properties of antimicrobial peptides rather than engage a specific molecular target, these experiments were aimed at defining the cellular consequences of FK20 exposure rather than identifying a single receptor or uptake pathway. The ability of FK20 RPM to associate with and penetrate *C. auris* cells was assessed using N-terminally 5(6)-carboxyfluorescein-labeled FK20 RPM. *C. auris* cells incubated with fluorescently labeled FK20 for 45 min and stained with Calcofluor White were visualized by confocal microscopy. Fluorescein-labeled FK20 (green) was detected within the boundaries of the Calcofluor White-stained cell wall (blue), as confirmed by Z-stack imaging and three-dimensional reconstruction (Fig. 3A; Movie S1). Importantly, fluorescein labeling did not impair FK20 antifungal activity, as labeled FK20 retained fungicidal potency comparable to unlabeled FK20, as assessed by CFU enumeration (Fig. 3B). Consistent with this retained activity, fluorescein-labeled FK20 induced slightly increased membrane permeabilization relative to unlabeled FK20, as reflected by enhanced propidium iodide uptake, a phenomenon previously reported for N-terminally modified antimicrobial peptides and attributable to augmented membrane interactions rather than a distinct mechanism of action (Fig. 3C) (48). To exclude nonspecific dye uptake or fluorescence artifacts, a series of specificity controls were included. Free fluorescein did not accumulate in *C. auris* cells under any condition tested, either when applied alone or when co-incubated with unlabeled FK20, indicating that intracellular fluorescence required peptide conjugation and was not driven by membrane damage-mediated dye entry (Fig. 3A). In contrast, fluorescein-labeled FK20 showed robust cellular association, consistent with its retained antifungal activity. Fluorescein-labeled IK10 and LK20 RPMs—peptides displaying only minimal antifungal activity—exhibited markedly reduced cellular association under identical labeling and imaging conditions (Fig. 3A). Flow cytometric quantification confirmed significantly higher uptake of FK20 relative to IK10 and LK20, demonstrating that intracellular accumulation correlates with antifungal potency rather than nonspecific membrane disruption or dye uptake (Fig. 3D). To investigate FK20-induced surface morphological changes, scanning electron microscopy (SEM) was performed on *C. auris* cells treated with 200 µg/mL of FK20 RPM stereoisomers (Fig. 4A). Homochiral RPMs (FK20 and dFdK20) induced pronounced surface disruption and extensive cellular aggregation compared to heterochiral FdK20 RPM and untreated controls. These findings indicate that homochirality enhances physicochemical interactions with the fungal surface, leading to more effective membrane destabilization. Membrane integrity was further assessed by measuring propidium iodide (PI) uptake using flow cytometry. FK20 treatment resulted in a concentration-dependent increase in PI-positive cells, confirming progressive membrane permeabilization (Fig. 4B). Similar results were observed using Ghost Dye, an amine-reactive viability dye (Fig. S2). Because RPM-induced membrane damage enables dye entry, intracellular fluorescence is expected to follow loss of membrane integrity rather than precede it, consistent with a membrane-disruptive mechanism of action.

Given that membrane-active antimicrobials often induce secondary cellular stress responses (49, 50), intracellular reactive oxygen species (ROS) production was measured using dihydrorhodamine 123 (DHR123) (Fig. 4C). FK20-treated *C. auris* exhibited significantly increased ROS levels relative to controls. In parallel, membrane depolarization was assessed using bis-(1,3-dibutylbarbituric acid) pentamethine oxonol [DiBAC_4_(5)], revealing a marked increase in fluorescence following FK20 exposure, indicative of collapse of membrane potential (Fig. 4D). Collectively, these data demonstrate that FK20 RPM exerts antifungal activity through a physicochemical mechanism dominated by membrane disruption, loss of membrane potential, ionic imbalance, and downstream oxidative stress, rather than through engagement of a specific intracellular target.

### FK20 RPM does not induce resistance in *C. auris* and exhibits collateral sensitivity in caspofungin-resistant strains

**Fig 5:**
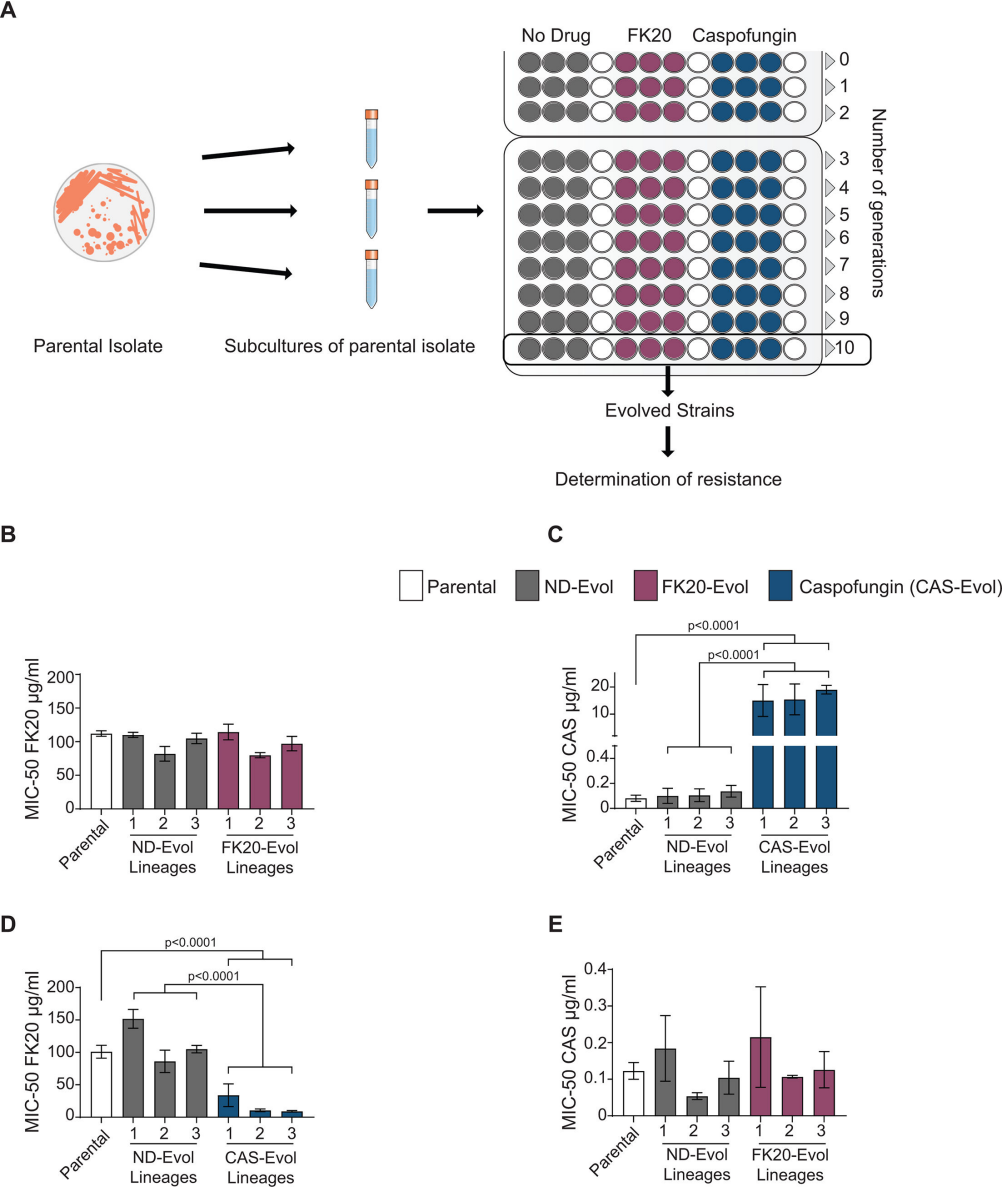
*In vitro* experimental evolution of *Candida auris*. (**A**) Schematic representation of the experimental evolution strategy. Parental *C. auris* cells were propagated under three conditions: without treatment, or in the presence of either FK20 or caspofungin to generate three independent evolved lineages after 10 sequential passages. Following evolution, growth profiles and resistance levels were determined by calculating MIC_50_ values for the parental, non-drug-evolved, and drug-evolved strains. Broth microdilution assays were performed to quantify growth by measuring optical density at OD _590_ across a range of increasing FK20 and caspofungin concentrations and determine MIC-50. Data are presented as MIC_50_ after 72 h of incubation under the respective drug conditions. (**B**) MIC_50_ values for FK20 in parental, non-drug-evolved, and FK20-evolved lineages. (**C**) MIC_50_ values for caspofungin in parental, non-drug-evolved, and caspofungin-evolved lineages. (**D and E**) Collateral sensitivity analysis of evolved strains. (**D**) FK20 MIC_50_ values for parental, non-drug-evolved, and caspofungin-evolved lineages. (**E**) Caspofungin MIC-50 values for parental, no-drug-evolved, and FK20-evolved lineages. Each data point represents the mean derived from three independent experiments performed in triplicate. Statistical significance was determined by one-way ANOVA with Tukey’s multiple comparisons test. Values without symbols are not significantly different from the control.

The rapid evolution of drug resistance in pathogens is a primary cause of treatment failure, often diminishing the efficacy of new antifungals shortly after market entry. Evaluating a drug candidate’s resistance potential is therefore critical. Our previous studies on the antibacterial activity of RPMs demonstrated that bacteria were unable to develop resistance, suggesting a promising low-resistance potential for this class of compounds (51, 52). To investigate the potential of *C. auris* to acquire resistance against FK20 RPM, *in vitro* experimental evolution was conducted following established protocols with minor modifications (53) (Fig. 5A). The FK20 RPM MIC_50_, defined as the drug concentration inhibiting growth by at least 50% relative to drug-free control, was first determined (Fig. S3A), resulting in values of 23.80 ± 7.09 µg/mL at 24 h, 54.16 ± 11.67 µg/mL at 48 h, and 104.10 ± 19.82 µg/mL at 72 h. Three parallel *C. auris* lineages were evolved in RPMI-1640 medium containing FK20 RPM (100 µg/mL, MIC_50_), caspofungin (2.0 µg/mL, MIC₅₀), or no drug (ND-Evol) through 10 serial passages, diluted 1:1,000 every 72 h, resulting in the emergence of three independently evolved strains for each condition. Evolved strains were then challenged with their respective antifungal agents, and growth kinetics were measured as optical density (OD) after 72 h to determine new MIC_50_ values, compared to parental and ND-Evol strains. FK20-evolved strains showed no significant change in the FK20 MIC_50_ relative to parental or ND-Evol strains (Fig. 5; Fig. S2B and C; Table 1). In contrast, caspofungin-evolved strains exhibited a marked increase in caspofungin MIC_50_ to >10 µg/mL (Fig. 5C; Fig. S2D and E). These findings demonstrate that while *C. auris* rapidly develops resistance to caspofungin within 10 passages, it fails to evolve resistance to FK20 RPM over the same timeframe, highlighting FK20’s potential to limit resistance development.

**TABLE 1:** MIC_50_ profiles of parental and evolved strains over time^*a*^

Strain	Lineage	MIC_50_ (µg/mL)					
		24 h		48 h		72 h	
		FK20	CAS	FK20	CAS	FK20	CAS
Parental		23.80 ± 7.09	0.08 ± 0.06	54.16 ± 11.67	0.10 ± 0.07	104.10 ± 19.82	0.10 ± 0.07
ND-Evol	1	28.85 ± 10.90	0.07± 0.04	73.69 ± 21.85	0.10 ± 0.08	130.90 ± 28.08	0.14 ± 0.13
	2	24.86 ± 15.14	0.10 ± 0.11	48.92 ± 7.14	0.06 ± 0.06	84.06 ± 22.47	0.08 ± 0.06
	3	23.63 ± 8.31	0.12 ± 0.08	60.93 ± 13.52	0.13 ± 0.08	104.94 ± 10.61	0.12 ± 0.07
FK20-Evol	1	17.00 ± 4.55	0.1 ± 0.09	54.59 ± 7.97	0.18 ± 0.22	114.40 ± 20.11	0.22 ± 0.24
	2	11.61 ± 2.12	0.12 ± 0.11	43.53 ± 8.74	0.20 ± 0.24	79.97 ± 6.63	0.11 ± 0.01
	3	17.39 ± 3.92	0.11 ± 0.11	47.15 ± 3.74	0.12 ± 0.10	97.14 ± 18.63	0.13 ± 0.09
CAS-Evol	1	6.09 ± 1.68	0.04 ± 0.01	14.28 ± 3.22	8.67 ± 3.79	33.91 ± 30.42	15.03 ± 8.33
	2	5.59 ± 0.34	0.09 ± 0.09	9.89 ± 2.04	6.89 ± 5.83	10.66 ± 3.56	15.46 ± 8.03
	3	7.28 ± 1.32	0.06 ± 0.04	9.14 ± 0.42	6.48 ± 6.30	9.12 ± 2.04	19.02 ± 2.26

^*a*^ Minimum inhibitory concentrations (MIC_50_ ± SEM) for FK20 and caspofungin were determined for the parental strain and experimentally evolved strains (ND-Evol, FK20-Evol, and CAS-Evol; three independent lineages per evolved condition) after 24, 48, and 72 h of incubation. Values represent the mean ± SEM from three independent experiments.

Collateral sensitivity, an evolutionary trade-off where resistance to one antifungal agent increases susceptibility to another (54), was assessed by comparing the resistance profiles of FK20-evolved and caspofungin-evolved *C. auris* strains when challenged with the reciprocal antifungal (caspofungin or FK20 RPM, respectively). FK20-evolved strains show no significant change in caspofungin MIC_50_ compared to parental or ND-Evol strains (Fig. 5E; Fig. S2H and I). In contrast, caspofungin-evolved strains exhibited a marked reduction in FK20 RPM MIC-50 (Fig. 5D; Fig. S2F and G), indicating collateral sensitivity to FK20 RPM. These findings suggest that while FK20 exposure does not alter caspofungin susceptibility, caspofungin resistance enhances susceptibility to FK20 RPM, a property with potential therapeutic applications.

### FK20 RPM and caspofungin are synergistic and inhibit *C. auris* biofilm formation

**Fig 6:**
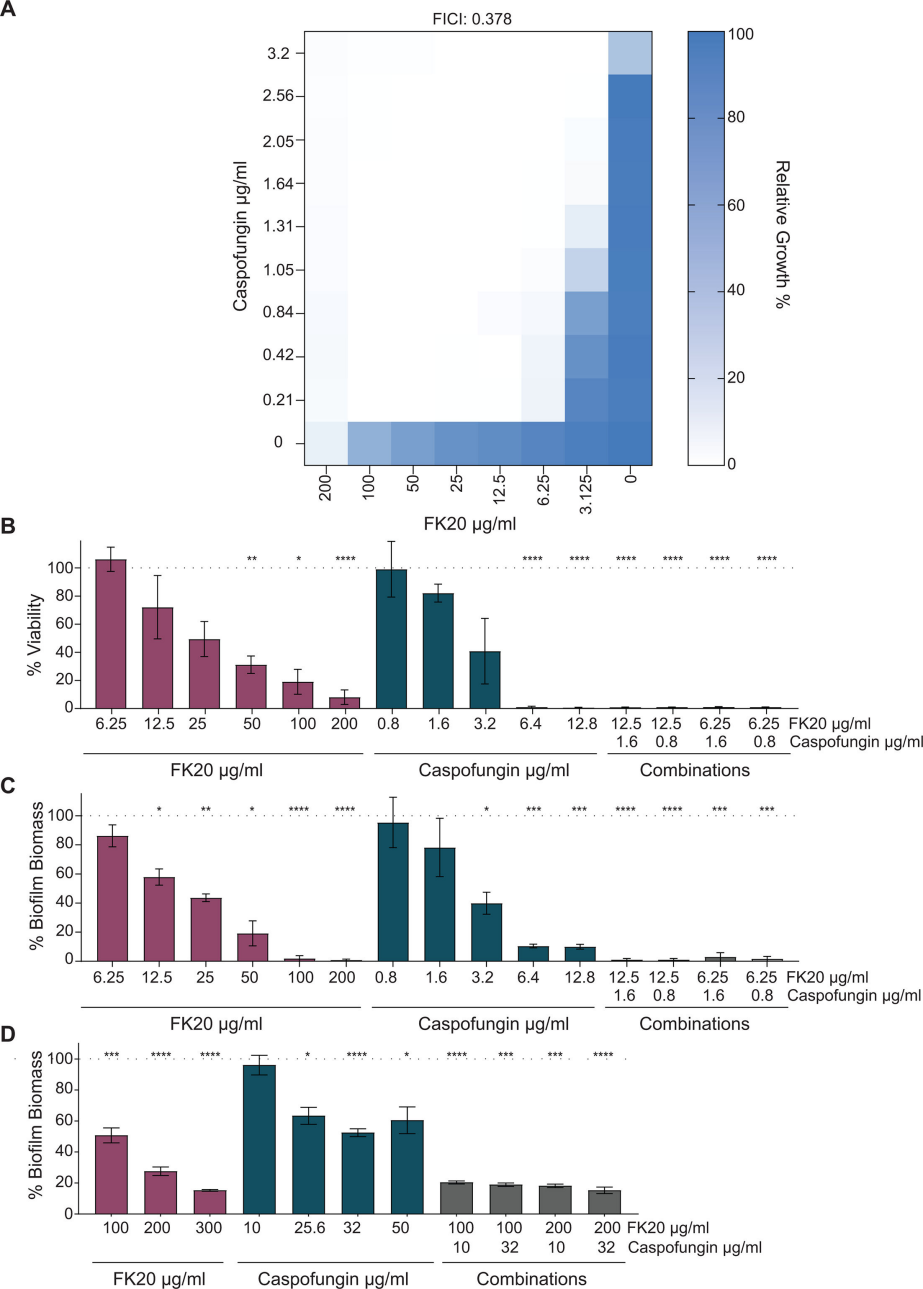
FK20 synergizes with caspofungin to exert fungicidal activity against *C. auris*, inhibit biofilm formation, and disrupt pre-formed mature biofilms. (**A**) Combined effects of FK20 and caspofungin on *C. auris* growth assessed by checkerboard assay. Fractional inhibitory concentration index values were calculated, and the heat map depicts percentage growth under various combinations of caspofungin and FK20 concentrations. (**B and C**) Inhibition of *C. auris* biofilm formation by FK20, caspofungin, or their combination, quantified after 24 h using the 3-(4,5-dimethyl thiazolyl-2)-2,5-diphenyl tetrazolium bromide assay (**B**) and crystal violet staining (**C**). FK20 and caspofungin were present in the growth medium from the start of the experiment. (**D**) Destruction of pre-formed mature *C. auris* biofilms following treatment with FK20, caspofungin, or their combination. *C. auris* cells (1 × 10^5^ cells/mL) were incubated in RPMI-1640 supplemented with L-glutamine (1%) and glucose (2%) and grown in a static culture for 24 h at 37°C to allow formation of mature biofilm. Planktonic cells were removed, and pre-formed, mature biofilms were washed with ultra-pure water. FK20, caspofungin, or their combinations were added at the indicated concentrations and incubated at 37°C for 24 h. Biofilm biomass was determined by crystal violet staining. Data represent mean percentage biomass or viability ± SEM of three biologically independent experiments performed with three technical replicates per condition. Significance levels are indicated as *P < 0.05, **P < 0.01, ***P < 0.001, and ****P < 0.0001.

Combination therapies leveraging collateral sensitivity can reduce resistance evolution, as mutations conferring resistance to one antifungal may increase susceptibility to another (55). Given the observed collateral sensitivity to FK20 RPM in caspofungin-resistant *C. auris* strains, the potential synergistic interaction between FK20 RPM and caspofungin was investigated. FK20 disrupts *C. auris* cell walls and membranes, while caspofungin inhibits the enzyme (1→3)-β-D-glucan synthase, compromising cell wall integrity (56). It was hypothesized that their combined treatment would induce synergistic cell wall damage and osmotic stress. A checkerboard assay was conducted, challenging *C. auris* cells with FK20 RPM (0–200 µg/mL) and caspofungin (0–3.2 µg/mL). The MIC_50_ values for FK20 RPM and caspofungin in combination were 3.125 and 1.05 µg/mL, respectively (Fig. 6A). The fractional inhibitory concentration index (FICI), calculated using established methods (57), yielded a value of 0.378, confirming a synergistic interaction between FK20 RPM and caspofungin.

Biofilm formation is a critical virulence factor in *Candida* species and plays a central role in *C. auris* persistence, immune evasion, and antifungal tolerance (58). Biofilm-associated phenotypes can be interrogated at distinct stages that reflect different clinical scenarios. Inhibition of biofilm formation models early intervention settings, such as initial host colonization, catheter insertion, or prophylactic antifungal exposure, where preventing adhesion and early biofilm maturation can limit persistence, dissemination, and the development of antifungal tolerance (59, 60). This is particularly relevant for *C. auris*, which readily colonizes skin and medical devices and serves as a reservoir for nosocomial transmission (61, 62). In contrast, assays targeting pre-formed mature biofilms address established infections, which are typically far more tolerant to antifungal therapy due to metabolic dormancy and extracellular matrix-mediated drug exclusion. For instance, caspofungin exhibits limited efficacy against *C. auris* biofilms, requiring concentrations (>32 µg/mL for 90% inhibition) that exceed safe clinical doses (58, 63). Therefore, assessing both biofilm prevention and activity against mature biofilms provides complementary and clinically relevant insights into antifungal efficacy.

Given the synergistic interaction between FK20 RPM and caspofungin, their combined potential to inhibit biofilm formation was evaluated. We first evaluated the ability of FK20 RPM and caspofungin, alone and in combination, to prevent biofilm formation. A biofilm inhibition assay was conducted by culturing *C. auris* cells in a polystyrene 96-well plate with varying concentrations of FK20 RPM, caspofungin, or their combinations. After 24 h, biofilms were quantified using 3-(4,5-dimethyl thiazolyl-2)-2,5-diphenyl tetrazolium bromide (MTT) and crystal violet assays (Fig. 6B and C). Individually, caspofungin and FK20 reduced biofilm formation in a concentration-dependent manner, achieving complete inhibition of biofilm viability, as determined by MTT assay, at 200 µg/mL FK20 and 6.4 µg/mL caspofungin, and complete inhibition of biofilm biomass as determined by crystal violet staining, at 100 µg/mL FK20 and 12.8 µg/mL caspofungin, respectively. Neither agent inhibited biofilm formation at low concentrations. In contrast, four combinations of FK20 RPM and caspofungin completely inhibited biofilm formation at concentrations 16-fold lower for FK20 and 8-fold lower for caspofungin than those required when each agent was used alone. Bliss independence analysis of biofilm formation inhibition, quantified by crystal violet staining, yielded positive Bliss scores ranging from 0.44 to 0.81, consistent with synergistic interactions, with the highest Bliss score observed at the lowest concentrations of both compounds. These findings highlight the potent synergistic efficacy of FK20 and caspofungin in preventing *C. auris* biofilm formation at clinically relevant concentrations.

Given the marked resistance of established *C. auris* biofilms to antifungal therapy, we next examined the activity of FK20 RPM against pre-formed (24 h) mature biofilms, both alone and in combination with caspofungin (Fig. 6D). Consistent with previous reports, caspofungin alone did not significantly reduce mature *C. auris* biofilm biomass at therapeutically relevant concentrations up to 10 µg/mL, with only partial biofilm reduction observed at higher, toxic concentrations (>25 µg/mL). In contrast, FK20 RPM exhibited substantial antibiofilm activity against mature biofilms as a single agent, resulting in dose-dependent reductions in biofilm biomass of approximately 49% at 100 µg/mL, 73% at 200 µg/mL, and 85% at 300 µg/mL.

Notably, the FK20–caspofungin combination displayed synergistic activity against mature biofilms at FK20 concentrations (100 µg/mL) that are twofold lower than its MIC in planktonic culture, when combined with caspofungin at 10 µg/mL, as determined by Bliss independence analysis (S_Bliss = 0.3). While synergy scores declined at higher drug concentrations, this observation is notable given that synergistic interactions against pre-formed *C. auris* biofilms are rarely observed, even for combinations that are highly synergistic against planktonic cells (64). Together, these data indicate that FK20 RPM retains potent antibiofilm activity against mature *C. auris* biofilms and can sensitize biofilm-embedded cells to caspofungin at otherwise ineffective concentrations.

### FK20 RPM demonstrates therapeutic potential in a murine candidiasis model

To evaluate the therapeutic potential of FK20 RPM, its cytotoxicity was assessed *in vitro* using RAW 264.7 murine macrophages, with IK20 RPM as a control. Cells were incubated with increasing concentrations of each RPM for 24 h, and viability was measured using the MTT assay (Fig. S3). The control 10-mer and 20-mer IK RPMs exhibited no cytotoxicity at concentrations up to 400 µg/mL. In contrast, FK20 RPM induced approximately 13% cytotoxicity at 200 µg/mL, a concentration effective against *C. auris*. At 400 µg/mL, twice its MIC, FK20 RPM exhibited pronounced cytotoxicity (59%) in macrophages, whereas IK20 and IK10 RPMs remained non-cytotoxic. These results indicate a concentration-dependent cytotoxic effect of FK20 RPM and suggest a therapeutic window in which antifungal activity is achieved with limited host-cell toxicity. To confirm FK20’s safety *in vivo*, mice received daily intramuscular injections of FK20 RPM (25 mg/kg of body weight), IK20 RPM (25 mg/kg of body weight, control), or ultra-pure water (UPW) for 4 days. The FK20 dosing regimen and route of administration were selected based on prior *in vivo* studies demonstrating efficacy, stability, effective drug distribution, and tolerability of FK20 random peptide mixtures in murine infection models. In these studies, FK20 administration via intramuscular or intravenous injection did not induce hemolysis, abnormal serum chemistry, or histopathological signs of toxicity and was well tolerated at doses equal to or exceeding those used here (45, 65). The present dosing strategy, therefore, reflects a literature-supported, conservative approach for early *in vivo* evaluation. On day 4, blood chemistry and hemolysis were analyzed (Fig. S4; Table S1). No significant differences were observed in hemolysis or blood chemistry profiles between FK20-treated and IK10 or UPW-treated cohorts, indicating FK20’s safety for therapeutic use. Although systemic FK20 concentrations were not directly measured in this study, prior work has demonstrated that FK20 random peptide mixtures are stable *in vivo* for at least 24 h following intramuscular or intravenous administration in mice (45, 65). This stability indicates resistance to host proteolytic degradation and supports biologically meaningful exposure during the treatment period.

**Fig 7:**
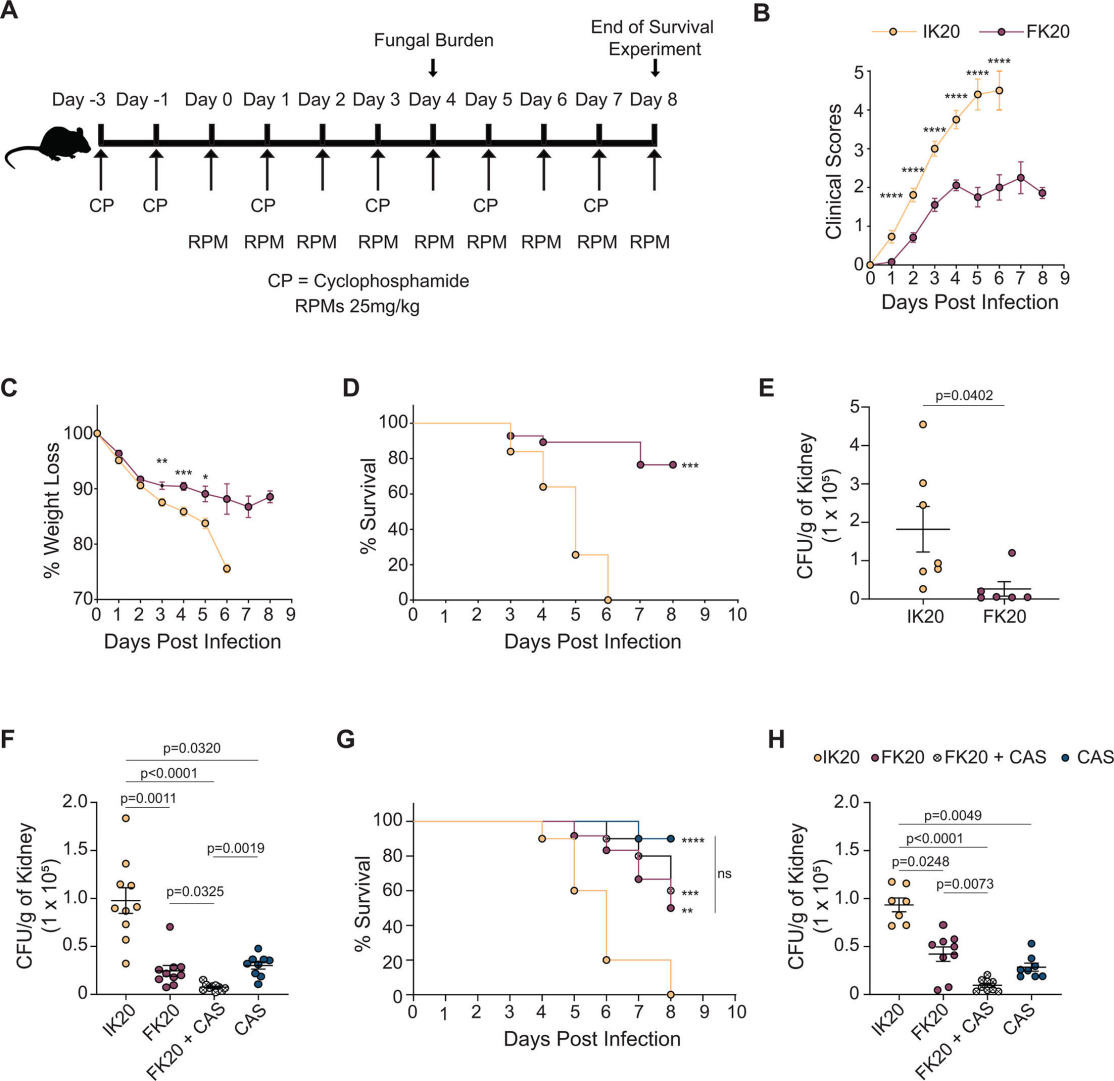
FK20 exhibits potent fungicidal activity in a murine model of systemic candidiasis. (**A**) Schematic overview of the experimental timeline for the murine systemic candidiasis model. C57BL/6 mice were immunosuppressed with cyclophosphamide and subsequently infected intravenously with 1 × 10^6^ CFUs of *C. auris*. Mice received daily intramuscular injections of FK20 or IK20 RPMs (25 mg/kg of body weight) throughout the study unless otherwise stated. (**B and C**) Disease severity assessed by semiquantitative clinical scores (**B**) and percentage weight loss (**C**). Clinical scores (0–5) were assigned based on assessments of fur condition, posture, activity, and lethargy, with higher scores indicating more severe illness. (**D**) Survival percentage of *C. auris*-infected mice treated with 25 mg/kg of body weight of FK20 or IK20 RPMs over a period of 8 days post-infection (*n* = 10 per group). (**E**) Fungal burden (CFU/g kidney tissue) on day 4 post-infection in mice treated with FK20 or IK20 RPMs. Data are presented as mean ± SEM (*n* = 7 for IK20, *n* = 6 for FK20). (**F**) Fungal burden in kidneys of mice treated with FK20 or IK20 RPMs (25 mg/kg of body weight), caspofungin (5 mg/kg of body weight), or their combination (25 mg/kg FK20 + 5 mg/kg caspofungin); *n* = 10 per group. (**G**) Corresponding percentage survival of mice under the same treatment regimens as panel **F** for a period of 8 days post-infection (*n* = 10 per group). (**H**) Fungal burden in kidneys of mice treated with reduced doses of FK20 or IK20 RPMs (15 mg/kg of body weight), caspofungin (3 mg/kg of body weight), or their combination; *n* = 10 per group. Data are expressed as CFU/g of kidney tissue. Statistical analysis: (**B, C, and E**) Student’s *t*-test (*P* < 0.05); (**D and G**) log-rank (Mantel–Cox) test; (**F and H**) one-way ANOVA with Tukey’s multiple comparisons test. Significance levels are indicated as **P < 0.01, ***P < 0.001, and ****P < 0.0001; ns, not significant.

To evaluate the *in vivo* efficacy of FK20 RPM, a neutropenic mouse model of invasive candidiasis was employed, involving intravenous *C. auris* challenge and assessment of treatment response via kidney fungal burden (66). Immunosuppression was induced with cyclophosphamide, followed by intravenous *C. auris* infection. Mice received daily intramuscular injections of FK20 RPM (25 mg/kg of body weight) or IK20 RPM (25 mg/kg of body weight, control), which lacks antifungal activity. Four days post-infection, disease severity, survival, weight loss, and kidney fungal burden were assessed (Fig. 7A through E). Compared to IK20-treated mice, FK20-treated mice exhibited significantly reduced clinical scores, increased survival rates, attenuated weight loss, and lower fungal burden. These findings demonstrate that FK20 RPM effectively mitigates acute *C. auris* infection *in vivo*, highlighting its therapeutic potential for systemic candidiasis. In addition to its antifungal efficacy, FK20 treatment was well tolerated *in vivo*. FK20-treated mice did not exhibit significant weight loss, abnormal behavior, or overt clinical signs of toxicity during the course of the experiment. Analysis of serum chemistry and hemolysis on day 4 revealed no significant differences between FK20-treated and control animals, indicating an absence of detectable acute toxicity at the administered dose. Together, these results demonstrate that FK20 random peptide mixtures exhibit *in vivo* antifungal activity against *C. auris* while remaining well tolerated under the conditions tested.

Given the observed *in vitro* synergy and collateral sensitivity between FK20 and caspofungin, we next assessed the efficacy of combination therapy *in vivo* (Fig. 7F through H). Neutropenic mice were intravenously infected with *C. auris* and treated daily with FK20 RPM (25 mg/kg of body weight), caspofungin (5 mg/kg of body weight), or the combination of both agents. At these doses, FK20 and caspofungin monotherapies each resulted in a substantial reduction in kidney fungal burden compared to control-treated mice (approximately 70% reduction). Combination treatment further decreased fungal burden (92% inhibition relative to control); however, this level of inhibition was consistent with the expected additive effect based on the individual drug activities, and Bliss independence analysis did not indicate a synergistic interaction under these conditions. To increase the dynamic range for detecting enhanced combination effects, additional experiments were performed using reduced drug doses (FK20, 15 mg/kg of body weight; caspofungin, 3 mg/kg of body weight). Under these conditions, FK20 monotherapy exhibited a modest reduction in efficacy (57% reduction compared to IK20-treated control), whereas caspofungin retained strong antifungal activity (approximately 70% reduction compared to IK20-treated control). Combination treatment resulted in 90% inhibition of fungal burden relative to control, closely matching the inhibition predicted from additive drug effects and yielding a Bliss score indicative of additive, rather than synergistic, activity. Notably, the high potency of each monotherapy under both dosing regimens limited the dynamic range available for resolving additive versus synergistic interactions *in vivo*.

Together, these findings establish FK20 as a potent antifungal agent against *C. auris*, exhibiting fungicidal activity *in vitro*, efficacy against biofilm formation and mature biofilms, and therapeutic activity in a murine model of systemic candidiasis. In addition, repeated exposure experiments failed to select for FK20-resistant *C. auris* populations under conditions that readily permitted resistance to conventional antifungals, indicating a high barrier to resistance development. FK20 displays collateral sensitivity with caspofungin and acts synergistically with this agent *in vitro*, resulting in enhanced antifungal activity. *In vivo*, both FK20 and caspofungin monotherapies significantly reduced fungal burden, and combination treatment further improved infection control, achieving near-complete clearance under the conditions tested. Collectively, these results support FK20 as a robust antifungal candidate with durable activity and potential utility both as a standalone therapy and in combination with echinocandins.

## DISCUSSION

The escalating threat of antimicrobial resistance, underscored by reports from the World Health Organization and the CDC, represents a global public health crisis (8, 67). While *C. albicans* remains prevalent, the rise of non-*albicans Candida* species, particularly multidrug-resistant *C. auris*, has shifted the epidemiology of candidiasis over the past decade, with *C. auris* classified as an urgent threat by the CDC due to high treatment failure rates (24). Current antifungals, including echinocandins, amphotericin B, and azoles, are limited by narrow spectra, toxicity, pharmacokinetic variability, and drug interactions. Echinocandins, despite favorable safety profiles, are susceptible to resistance in *C. auris*, while amphotericin B and azoles are constrained by toxicity and drug-drug interactions, respectively (68, 69). These limitations, coupled with emerging resistance, necessitate novel antifungal agents with unique mechanisms and robust resistance profiles.

Our study demonstrates that FK20 RPM, a homochiral L-phenylalanine–L-lysine 20-mer, exhibits broad-spectrum antifungal activity with species-specific potency across major human fungal pathogens, including *Candida* spp., *C. neoformans*, and *A. fumigatus*, with particularly high activity against *C. auris*. RPMs are explicitly designed to recapitulate key physicochemical features of AMPs—cationic charge, amphipathicity, and short chain length—while avoiding reliance on a single defined sequence or tertiary structure. Accordingly, FK20’s mode of action is best understood as a collective, stochastic physicochemical attack on fungal membranes rather than a target-specific interaction. Multiple orthogonal readouts support this model: FK20 exposure induced extensive cell surface disruption (SEM), concentration-dependent membrane permeabilization (PI and Ghost Dye uptake), membrane depolarization [DiBAC_4_(5)], and oxidative stress (DHR123), all hallmarks of membrane-active antimicrobial agents (Fig. 4; Fig. S2). Fluorescence microscopy and flow cytometry further demonstrate FK20 association with and internalization into *C. auris* cells (Fig. 3); however, this internalization is most parsimoniously interpreted as a consequence of membrane compromise rather than an active uptake mechanism, a distinction well established for AMPs and membrane-disrupting antibiotics (70, 71). Consistent with this interpretation, fluorescein-labeled FK20 retained full antifungal activity and showed robust cellular association, whereas fluorescein-labeled control RPMs with minimal antifungal activity (IK10 and LK20) and free fluorescein failed to accumulate in viable fungal cells. Together, these findings support a selective, physicochemically driven interaction with fungal membranes.

The preferential activity of FK20 against fungal cells likely arises from fundamental differences in lipid composition, membrane biophysics, and surface electrostatics between fungal and mammalian membranes, together with the intrinsic physicochemical properties of RPMs. Fungal plasma membranes are enriched in negatively charged phospholipids such as phosphatidylserine and phosphatidylinositol, which are more accessible at the outer leaflet than in mammalian membranes dominated by zwitterionic lipids (72, 73). This generates strong electrostatic attraction for cationic, amphipathic peptides like FK20, enabling preferential fungal membrane binding independent of receptors or active uptake (74). Sterol composition further enhances selectivity. Ergosterol-rich fungal membranes differ from cholesterol-containing mammalian membranes in packing, fluidity, and elasticity, rendering them more susceptible to peptide insertion and destabilization, while cholesterol confers resistance to membrane thinning and curvature stress in mammalian cells (75, 76). RPMs amplify these intrinsic differences through a collective, stochastic mode of action. Consisting of thousands to millions of short, amphipathic peptides lacking fixed tertiary structure, RPMs insert in multiple orientations and generate distributed nanoscale disruptions rather than discrete pores, preferentially destabilizing membranes with high negative surface potential and lower elastic moduli—features characteristic of fungal membranes.

This framework also explains the observed species-specific differences in susceptibility. FK20 displayed reduced potency against *A. fumigatus* compared with both *Candida* spp. and *C. neoformans* (Fig. 2A), consistent with the exceptionally high melanin content of *A. fumigatus* conidia (~5%–10% of cell wall dry weight) relative to *Candida* spp. and *C. neoformans* (~1%). Melanin is a negatively charged, hydrophobic polymer that acts as a physical and chemical barrier by sequestering cationic peptides, limiting their access to the plasma membrane, and scavenging reactive oxygen species, thereby attenuating AMP-mediated killing. These properties are well documented to reduce the activity of membrane-active antimicrobial peptides and support a physicochemical, rather than target-specific, mode of action (77, 78). In contrast, *C. neoformans* was highly susceptible not only to FK20 but also to IK20 (Fig. 2A), despite the lower hydrophobicity of isoleucine compared with phenylalanine. This heightened sensitivity likely reflects differences in membrane composition, particularly the lower ergosterol content of *C. neoformans*, which may render its plasma membrane more vulnerable to even moderately hydrophobic RPMs. Accordingly, IK20—while minimally fungicidal against *Candida* spp. and *A. fumigatus*—retains appreciable activity against *C. neoformans*, illustrating how subtle changes in RPM hydrophobicity can selectively tune membrane interactions across fungal species.

Rather than representing a limitation, the absence of a specific molecular target constitutes a major therapeutic advantage, particularly against pathogens such as *C. auris* that rapidly acquire target-based drug resistance. Unlike many natural AMPs, which may rely on defined secondary structures or stereospecific interactions, RPMs comprise thousands to millions of related but non-identical sequences. This intrinsic heterogeneity eliminates a conserved binding epitope, preventing fungi from evolving resistance through receptor modification or sequence-specific antagonism. Importantly, this generalized membrane-targeting mechanism imposes a high barrier to resistance evolution. Fungi cannot substantially alter membrane charge, lipid composition, or ergosterol content without incurring severe fitness costs.

Consistent with this principle, a particularly notable finding of this study is the striking asymmetry in resistance evolution and cross-susceptibility between FK20 RPM and caspofungin (Fig. 5D and E). Under identical experimental evolution conditions, *C. auris* rapidly acquired high-level resistance to caspofungin, with reduced MIC_50_, consistent with extensive clinical and experimental evidence demonstrating the low genetic barrier to echinocandin resistance (79–81). In contrast, prolonged exposure to FK20 failed to produce any detectable increase in FK20 MIC, indicating an exceptional inability of *C. auris* to adapt to this compound over multiple passages. Beyond the absence of resistance, caspofungin-resistant lineages exhibited pronounced collateral sensitivity to FK20, whereas FK20-evolved strains retained full susceptibility to caspofungin. The observed collateral sensitivity of caspofungin-resistant *C. auris* strains to FK20 RPM is consistent with known consequences of echinocandin resistance. Resistance-associated alterations in β-1,3-glucan synthesis and compensatory cell wall remodeling are expected to perturb cell wall–membrane coupling and increase membrane vulnerability (82–84), thereby sensitizing cells to membrane-disrupting agents such as FK20. Such unidirectional collateral sensitivity is uncommon among antifungal agents and has important therapeutic implications. The finding that caspofungin resistance enhances susceptibility to FK20 suggests a built-in evolutionary constraint: selection for resistance to one agent actively increases vulnerability to the other. In a combination setting, this creates opposing selective pressures that disfavor the stable emergence of caspofungin resistance, as resistant variants are preferentially eliminated by FK20. Drug pairs exhibiting this resistance–sensitivity trade-off are therefore considered particularly robust, as they both delay resistance evolution and maintain efficacy against resistant subpopulations.

Importantly, combination therapies are predicted to be most effective when such evolutionary constraints coincide with true pharmacological synergy. While collateral sensitivity limits the evolutionary escape routes available to the pathogen, synergy ensures enhanced killing at lower drug concentrations across multiple physiological states. In this context, our finding that FK20 and caspofungin not only exhibit unidirectional collateral sensitivity but also act synergistically positions this combination as an especially attractive therapeutic pairing (Fig. 6). This dual evolutionary–pharmacological advantage provides a strong rationale for the FK20–caspofungin combination and underpins the enhanced activity observed in planktonic cultures, during biofilm formation, and against established biofilms. Mechanistically, this synergy likely reflects complementary modes of action: FK20 disrupts fungal cell walls and membranes through physicochemical interactions, while caspofungin inhibits (1→3)-β-D-glucan synthase, compromising cell wall biosynthesis. Such convergent envelope damage, which exploits fundamental biophysical vulnerabilities rather than single molecular targets, is a recurring theme in successful antimicrobial combination therapies (85, 86). Consistent with this model, FK20–caspofungin combinations enabled complete inhibition of *C. auris* biofilm formation at concentrations 16- and 8-fold lower than the individual inhibitory concentrations of FK20 and caspofungin, respectively (Fig. 6B and C). Biofilm prevention assays capture early stages of fungal adhesion and community establishment, a clinically relevant window in which intervention may avert the development of highly drug-tolerant biofilm structures on host tissues or medical devices. Importantly, biofilm-associated phenotypes represent distinct biological states, with profound implications for antifungal susceptibility. While biofilm prevention assays model early stages of biofilm development, assays using pre-formed biofilms interrogate activity against highly structured, metabolically heterogeneous, and drug-tolerant fungal communities. Mature *C. auris* biofilms are particularly recalcitrant to antifungal therapy, often requiring caspofungin concentrations exceeding clinically achievable levels (>32 µg/mL for ~90% inhibition), a limitation consistently reported for echinocandins and other antifungal classes (79). In this context, our new data demonstrating that FK20 alone significantly inhibits pre-formed *C. auris* biofilms—and retains moderate but measurable synergy with caspofungin at sub-MIC concentrations—are particularly noteworthy. Unlike many standard antifungals, whose activity is largely restricted to planktonic or early biofilm stages, FK20 exhibits intrinsic antibiofilm activity against established biofilms, a phenotype rarely observed for azoles or echinocandins and more characteristic of certain antimicrobial peptides (58, 87, 88).

Several features of random peptide mixtures may underlie this activity. The extracellular matrix of fungal biofilms is highly negatively charged, favoring electrostatic accumulation of cationic RPMs (89, 90). In addition, the intrinsic sequence diversity of RPMs likely limits proteolytic inactivation, as no single protease can efficiently degrade the entire mixture. RPM-mediated membrane permeabilization and ion imbalance may further enable killing of metabolically dormant biofilm-embedded cells, which are largely tolerant to drugs targeting active biosynthesis (91, 92). Finally, resistance mechanisms based on matrix shielding or reduced penetration may be less effective against a heterogeneous, multi-sequence attack that operates through collective physicochemical interactions rather than a single molecular target.

FK20’s therapeutic potential is further supported by its safety and efficacy in an early preclinical, proof-of-concept setting. *In vitro,* FK20 exhibited minimal cytotoxicity to RAW 264.7 macrophages at 200 µg/mL—corresponding to twice the MIC required for *C. auris* inhibition—whereas cytotoxic effects were observed only at higher concentrations (400 µg/mL), indicating a therapeutic window between antifungal activity and host cell toxicity (Fig. S4). *In vivo*, FK20 (25 mg/kg of body weight) showed no adverse effects on blood chemistry or hemolysis in mice (Fig. S5; Table S1), addressing common toxic liabilities associated with cationic antimicrobial peptides, including membrane damage and red blood cell lysis, and supporting its overall tolerability at the administered dose. This dosing regimen and route of administration were selected based on prior *in vivo* studies demonstrating that FK20 RPMs are well tolerated in mice, with no evidence of hemolysis, abnormal serum chemistry, or histopathological toxicity following intramuscular or intravenous administration (45, 65). Although systemic FK20 concentrations and formal PK/PD relationships were not determined here, FK20 peptide mixtures have previously been shown to be stable *in vivo* for at least 24 h following administration in mice, indicating resistance to host proteolytic degradation and supporting sustained exposure during treatment.

In a neutropenic mouse model of candidiasis, FK20 significantly reduced fungal burden, clinical scores, and weight loss and increased survival compared to IK20-treated controls (Fig. 7B through E), demonstrating *in vivo* antifungal efficacy in a relevant model of systemic infection. These findings are consistent with the potent fungicidal activity observed *in vitro* and further validate FK20 as a promising candidate for the treatment of invasive *Candida* infections.

Given the pronounced *in vitro* synergy and collateral sensitivity observed between FK20 and caspofungin, we next explored the therapeutic potential of combination treatment *in vivo* (Fig. 7F through H). At the doses tested, both FK20 and caspofungin monotherapies were already highly effective, each producing substantial reductions in kidney fungal burden. Combination therapy resulted in a further decrease in fungal burden relative to either monotherapy alone; however, this additional reduction was quantitatively consistent with the level of inhibition predicted from additive drug effects, as assessed by Bliss independence analysis. Similar results were obtained when drug doses were reduced to increase the dynamic range for detecting enhanced combination effects, as caspofungin retained strong activity and FK20 remained potently antifungal. Under these conditions, combination treatment again achieved fungal burden reductions closely matching additive expectations, without evidence for *in vivo* synergy. These findings suggest that the high intrinsic potency of each agent in this acute infection model may impose a ceiling effect that limits the ability to discriminate between additive and synergistic interactions *in vivo*, a challenge that has been noted in other systemic antifungal combination studies (93–96). Importantly, the absence of detectable *in vivo* synergy under these conditions does not negate the therapeutic relevance of the FK20–caspofungin combination. Combination therapy consistently achieved near-maximal fungal clearance and may provide advantages in clinical contexts not fully captured by short-term fungal burden measurements, such as mitigation of resistance emergence, enhanced efficacy against heterogeneous fungal populations, or improved outcomes in infections caused by echinocandin-resistant isolates. Indeed, the robust *in vitro* synergy observed across multiple assays, together with the failure to select FK20-resistant *C. auris* under prolonged exposure, supports the rationale for further investigation of FK20-based combination regimens in models explicitly designed to probe resistance suppression and long-term treatment outcomes.

The scope of the *in vivo* evaluation presented here is consistent with prevailing practices in early-stage antimicrobial peptide research. Numerous AMP studies advance candidates into murine infection models based on a combination of *in vitro* cytotoxicity and hemolysis assays, together with limited *in vivo* safety readouts such as serum chemistry or short-term tolerability, without comprehensive pharmacokinetic or dose–exposure analyses (97, 98). Within this framework, the current study aligns with accepted standards by demonstrating *in vivo* efficacy while ruling out overt acute toxicity using safety endpoints that are directly relevant to the known liabilities of cationic, membrane-active peptides.

A limitation of the present study is that survival was monitored for up to 8 days post-infection, reflecting the use of a high-inoculum intravenous candidiasis model associated with rapid disease progression. Within this ethically approved timeframe, FK20 treatment (alone or in combination) resulted in near-complete fungal clearance by day 4 post-infection, as assessed by both CFU enumeration and qPCR-based measurements. These findings provide strong evidence for *in vivo* antifungal efficacy, while longer-term outcome studies and infection models optimized to interrogate combination effects will be of interest in future work.

In conclusion, FK20 RPM represents a mechanistically distinct antifungal strategy against *C. auris*, combining potent cell envelope-directed activity with a markedly reduced propensity for resistance development and favorable interactions with existing antifungals. FK20’s ability to exploit collateral sensitivity in caspofungin-resistant strains, together with its robust synergy with caspofungin in planktonic cells and measurable activity against mature biofilms, underscores its promise as a component of combination therapies aimed at overcoming both intrinsic and acquired antifungal resistance. Unlike conventional target-specific antifungals, FK20 retains activity across multiple fungal physiological states, including drug-tolerant biofilm-associated populations, a feature that is increasingly recognized as critical for durable therapeutic success. Importantly, FK20 demonstrated efficacy *in vivo* without overt toxicity, supporting the translational feasibility of random peptide mixtures as antifungal agents. Taken together, our findings suggest that FK20 and related RPMs may constitute a broadly applicable antimicrobial platform, particularly well-suited for infections characterized by biofilm formation, high tolerance, and rapid resistance emergence. Future studies should focus on defining optimal dosing and formulation strategies, expanding evaluation across diverse fungal pathogens and biofilm architectures, and assessing the performance of FK20-based combinations in advanced preclinical models. Such efforts will be essential to determine whether the unique properties of RPMs can be harnessed to complement or extend the current antifungal armamentarium in the clinic.

## MATERIALS AND METHODS

### Peptide synthesis

Peptides were synthesized by a standard Fmoc-based solid-phase peptide synthesis on Rink Amide resin (substitution 0.6 mmol/g), using a peptide synthesizer (Liberty Blue). Amino acids were diluted with dimethylformamide (DMF) to a final concentration of 0.2 M. Before coupling execution, four equivalents of amino acids were activated in DMF using four equivalents of DIC and 8 equivalents of Oxyma. Fmoc deprotection was conducted by 20% piperidine in DMF. At the end of the synthesis, the peptide mixtures were cleaved from the resin by adding a solution containing 95% trifluoroacetic acid (TFA), 2.5% double-distilled water (DDW), and 2.5% triisopropylsilane and stirring for 3 h. The mixture was then filtered, and the peptides were precipitated by the addition of 40 mL cold diethyl ether to the TFA solution and centrifuged. The supernatant was then removed, and the peptide pellet was dried, dissolved in 20% acetonitrile in DDW, frozen with liquid nitrogen, and lyophilized. The synthesis was validated by MALDI-TOF mass spectrometry.

### Cells and reagents

Mouse macrophage-like RAW 264.7 cells were cultured in DMEM medium supplemented with 10% fetal bovine serum, 1% glutamine, and 100 units/mL penicillin/streptomycin and maintained at 37°C in 5% CO_2_. Toxicity assay was done using 3-(4,5-dimethyl thiazolyl-2)-2,5-diphenyl tetrazolium bromide assay.

### *C. auris* killing assay

An overnight starter of *C. auris* was prepared in 5 mL of YPD medium and grown at 37°C with shaking at 200 rpm. The starter was washed three times with PBS. Cells were counted using a hemocytometer and resuspended in PBS. A total of 10^6^ cells/mL were incubated with 50 or 200 µg/mL of each RPM separately for 45 min at 37°C with shaking at 200 rpm in PBS. The microbial burden was calculated after dilutions and plating each sample on YPD agar plates.

### Scanning electron microscopy imaging of *C. auris*

An overnight starter culture of *C. auris* was grown in YPD at 37°C with shaking at 200 rpm. Cells were harvested and washed three times with PBS, counted with a hemocytometer, and diluted to a final concentration of 5 × 10^6^ cells/mL. Cell suspensions were incubated with 200 µg/mL of each RPM in PBS for 45 min at 37°C with shaking at 200 rpm. This was followed by additional washes with PBS. *C. auris* cells were resuspended in 4% glutaraldehyde and fixed for 1 h at room temperature. Fixed cells were washed with PBS, and 30 µL of each sample was placed above and below 0.5 mm pre-coated poly-L-lysine glass slides and incubated for 1 h at room temperature in a humid chamber to promote cell adhesion.

Samples were dehydrated through a graded ethanol series of increasing concentrations and then transferred to small porous baskets in ethanol to allow cells to reach critical point drying (Quorum K850) using liquid CO_2_. Ethanol was replaced by 100% liquid CO_2_ at 5°C–10°C, and the chamber was heated to 35°C so that at 31°C, liquid CO_2_ passed into the gas phase, and the resulting pressure was slowly released.

Dried samples were mounted on metal stubs and sputter-coated with an Au/Pd alloy at a ratio of 80:20 using a Q150T ES coater. Micrographs were acquired on a JEOL JSM-7800F field-emission scanning electron microscope at the Faculty of Agriculture, Food, and Environment, The Hebrew University of Jerusalem, operated in high-vacuum mode at 20 kV using a secondary electron detector.

### Mice and animal care

Eight-week-old C57BL/6JOlaHsd female mice (18–20 g) were purchased from Envigo, Israel. Mice were housed under specific pathogen-free conditions in groups of five in individually ventilated cages with unrestricted access to food and water.

### Animals and dosing rationale

The FK20 dosing regimen and route of administration were selected based on previously published *in vivo* studies demonstrating efficacy and tolerability of FK20 random peptide mixtures in murine infection models. In these studies, FK20 administration via intramuscular or intravenous injection did not induce hemolysis, abnormal serum chemistry, or histopathological signs of toxicity (45, 65). The dose used in the present study is equal to or lower than doses previously shown to be safe and effective against bacterial pathogens *in vivo*. Animals were monitored daily for body weight, behavior, and clinical signs of distress throughout the experiment.

### Murine model of systemic candidiasis

For toxicity studies, mice (cohorts of five) were injected intramuscularly once daily with FK20 or IK10 (25 µg/g of body weight), or with sterile ultra-pure water as a control. Mice were euthanized on day 5, and blood was collected by tail vein sampling. Blood chemistry analyses were performed by a certified veterinary diagnostic laboratory at the University Veterinary Hospital in Rishon LeZion, Israel. Blood chemistry was assessed in unfasted mice; therefore, glucose measurements may reflect procedural stress and were not used to infer basal metabolic status. To evaluate the hemolytic activity of FK20, 200 µL of blood was transferred to a 96-well plate and centrifuged at 1,000 × *g* for 10 min at 4°C. The plasma supernatant was then transferred to a fresh 96-well plate and diluted 1:30 (vol/vol) in PBS. Hemoglobin release was quantified spectrophotometrically by measuring absorbance at 540 nm. Plasma from control mice treated with 2% Triton X-100 served as a positive hemolysis control. These endpoints were selected to detect common toxic liabilities associated with cationic antimicrobial peptides, including membrane damage, red blood cell lysis, and organ injury.

Mice were immunosuppressed by intraperitoneal injection of 0.2 mL of cyclophosphamide in PBS at a concentration of 150 mg/kg of body weight 3 days before infection, followed by 100 mg/kg of body weight 1 day before infection and 2 days post-infection. Subsequent injection of cyclophosphamide (100 mg/kg of body weight) was administered at 2-day intervals following infection in the survival experiment. Infection inoculum was obtained by sub-culturing *C. auris* on agar plates grown at 37°C for 24 h. Single colonies of *C. auris* were resuspended in PBS and adjusted to specified concentrations. Immunosuppressed mice were infected with 1 × 10^6^ CFUs in 0.1 mL of PBS via the lateral tail vein injection. Treatment of mice with 25 µg/g of body weight of each RPM was initiated approximately 2 h after infection and repeated daily throughout the course of the experiment. Following infection, the health status and survival of mice were monitored daily. Clinical scores were assigned to each mouse to evaluate disease severity using disease severity indices obtained from observations of mouse activity, grooming, lethargy, and posture. The following parameters were included: fur, coat, and posture (normal, 0; fur mildly ruffled, 1; fur strongly ruffled, 2; fur strongly ruffled and hunched posture, 3; mild lethargy, 4; and severe lethargy, 5). The maximum possible score was 5. Mice were humanely sacrificed when they reached the humane endpoints defined as a clinical score of ≥4, or ≥20% loss of baseline body weight. The survival curves were analyzed by the log-rank (Mantel-Cox) test using GraphPad Prism software version 8.00 (GraphPad Software, USA). Mice were sacrificed, and the excised kidneys were weighed, followed by homogenization using GentleMACS Dissociator (Miltenyi Biotec). Subsequently, 100 µL of organ homogenate was used in the determination of CFUs following a 1:10,000 dilution.

### Determination of MIC

Minimum inhibitory concentration assays for *C. auris* B11117 strains were performed for FK20 and caspofungin according to the European Committee on Antibiotic Susceptibility Testing (EUCAST) guidelines for the determination of MIC using broth microdilution method (58, 59). Single colonies were obtained for *Candida auris* B11117 strains streaked on Sabouraud Dextrose Agar (40 g/L dextrose and 10 g/L peptone, at pH 5.6) plates at 37°C for 24 h. Approximately 1 × 10^5^ *C. auris* cells were incubated in 200 µL of filter-sterilized RPMI-1640 culture medium (Sigma), supplemented with 1.8% glucose to reach a final concentration of 2% and 0.03% L-glutamine at pH 7.0. The growth medium was supplemented with serial dilutions of antifungal agents 0–400 µg/mL of FK20 RPM and 0–64 µg/mL of caspofungin in different 96-well microtiter plates. Cells were incubated in 96-well microtiter plates at 37°C, and MIC was determined spectrophotometrically after 28, 48, and 72 h using Tecan Infinite M Plex plate reader (Neotec Bio, Israel).

### Drug combination assay and determination of fractional inhibitory concentration

The combined efficacy of caspofungin and FK20 was determined *in vitro* using a checkerboard assay, followed by the calculation of the FICI. Caspofungin and FK20 were added individually at a volume of 50 µL per well in fourfold strength, yielding a final concentration of 0–3.2 µg/mL for caspofungin and 0–2,000 to 200 µg/mL for FK20. The yeast inoculum (1 × 10^5^ cells per well) was added to the plates. The plates were then incubated at 37°C for a duration of 72 h. The antifungal combination activities of caspofungin and FK20 were assessed based on their FICI. The FICI was derived from the minimum inhibitory concentration (MIC_50_), representing the inhibitory concentration at which 50% of the cell population is killed by the effect of the drug combination. The FICI was determined by the following formula: $\;index=FIC_{A}+FIC_{B}=\frac{\left[A\right]}{MIC_{A}}+\frac{\left[B\right]}{MIC_{B}}$, where [A] and [B] are concentrations of drugs A and B and MIC_A_ and MIC_B_ are their respective MIC values for *C. auris*. Results were interpreted as follows: synergy (FICI ≤ 0.5), partial synergy (0.5 < FICI ≤ 0.75), additive (0.75 < FICI ≤ 1), indifferent (1 < FICI ≤ 4), and antagonistic (FICI > 4).

### Biofilm formation and quantification

In a 96-well microtiter plate, 200 µL of *C. auris* cells at a concentration of 10^6^ CFU/mL were cultured in RPMI-1640 medium, supplemented with L-glutamine (1%) and glucose (2%) for 24 h at 37°C together with different concentrations of FK20, caspofungin, or combinations that were tested to study their effects on biofilm formation. Inhibition of biofilm formation was assessed using a crystal violet assay and an MTT assay. Planktonic cells were removed; biofilms were washed with UPW and then stained with 125 µL of 0.1% crystal violet for 15 min. Excess stain was washed off, and the biofilms were air-dried for 4 h at 25°C. Finally, 125 µL of 30% acetic acid was added to each well, and biofilm biomass was quantified at 595 nm using a microplate reader (Tecan Infinite M200). Biofilm viability was assessed using the MTT assay. In this method, each well was loaded with 50 µL of MTT reagent (1.5 mg/mL), and the plate was incubated for 1.5 h at 37°C. Subsequently, 150 µL of dimethyl sulfoxide was added, and the plate was incubated for 10 min at 30°C with agitation at 180 rpm. The absorbance was then measured at 595 nm using a TECAN Infinite plate reader.

To quantify the ability of FK20 to disrupt pre-formed mature biofilms, *C. auris* cells (1 × 10^5^ cells/mL) were grown in RPMI-1640 medium, supplemented with L-glutamine (1%) and glucose (2%) for 24 h at 37°C to allow formation of mature biofilms. Planktonic cells were discarded, and mature biofilms were washed with PBS. FK20, Caspofungin, and their combinations at specified concentrations were added to each well and incubated at 37°C for 24 h. Each well was washed twice with UPW, and biofilm biomass was determined using the crystal violet assay as described above.

### *In vitro* experimental evolution

*In vitro* experimental evolution of *Candida auris* was performed according to the protocol published in reference 45 with minor modifications. Briefly, *C. auris* B11117 was independently passaged through 100 µg/mL of FK20 RPM and 2 µg/mL of caspofungin. *C. auris* was also passaged simultaneously without exposure to any antifungal drug. The concentration of FK20 RPM selected for the experimental evolution was equivalent to the predetermined MIC_50_ of the parental *C. auris* B11117 strain. The experimental evolution was initiated by generating three independent replicates of *C. auris* B11117 as the parental strain. Cultures were streaked from frozen parental stocks onto Sabouraud dextrose agar (40 g/L dextrose and 10 g/L peptone, at pH 5.6) plates and incubated overnight at 37°C. Each parental replicate was generated from randomly selected single colonies. Subcultures were made in YPD liquid medium and incubated overnight at 37°C at 100 rpm. The overnight culture was diluted 1:1,000 in RPMI-1640 (supplemented with 1.8% glucose to reach a final concentration of 2% glucose and 0.03% L-glutamine at pH 7.0) to a final volume of 1 mL. RPMI-1640 medium was supplemented with 100 µg/mL of FK20 and 2.5 µg/mL of caspofungin for replicates evolved with FK20 and caspofungin, respectively, and supplemented RPMI-1640 medium for replicates evolved without drugs in 96-deep well plates and incubated at 37°C for 72 h. From cultures of each replicate, 100 µL of each replicate was frozen in 25% glycerol as parental replicates at time zero. Precautions were taken to minimize contamination while keeping environmental conditions similar. Plates were sealed with gas-permeable membranes to allow gas exchange and maximum growth. The 96-deep well plate was incubated at 37°C without shaking to mimic static growth used in clinical resistance assays. Plates were incubated in plastic trays lined with wet paper towels to minimize evaporation. Following incubation for 72 h, individual wells were mixed by pipetting and diluted 1:10 into fresh RPMI-1640 supplemented with or without drugs. In total, 10 transfers were carried out, and 100 µL of evolved replicate cultures was frozen in duplicates in 25% glycerol for each transfer until after the 10^th^ transfer and maintained at −80°C.

### Determination of resistance for evolved strains

Initial susceptibility of all parental strains was tested using broth microdilution liquid assays to determine the MIC_50_, that is, the concentration of antifungal agent at which 50% of the cell population is eliminated. The liquid assay experiment was carried out according to cell dilution regulations of the EUCAST guidelines with some modifications (58, 59). Briefly, RPMI incubated at 37°C was used as the base medium, and growth was determined as absorbance of cell densities at OD_590_ at 24, 48, and 72 h post-inoculation. MIC_50_ was numerically calculated as the concentration at which a 50% decrease in turbidity was determined spectrophotometrically.

### Viability staining

To assess the fungicidal activity of FK20, approximately 1 × 10^6^ cells/mL were incubated with varying concentrations of FK20, ranging from 0 to 400 µg/mL for 45 min at 37°C with continuous shaking. Subsequently, treated cells were washed with PBS and resuspended with either propidium iodide (1:25 dilution of 0.5 mg/mL, BioLegend) or Ghost Dye (1:500 dilution, Tonbo Biosciences) viability stain for 20 min. Residual viability dyes were washed with PBS, and cells were resuspended in PBS for analysis of fluorescence by flow cytometer.

### Reactive oxygen species detection

Approximately 1 × 10^6^ cells/mL were incubated with 200 µg/mL of FK20 for 45 min at 37°C with continuous shaking at 200 rpm. Treated and untreated cells were washed with PBS and resuspended with 10 µM dihydrorhodamine 123 (Cayman Chemical) to detect intracellular ROS by staining for 20 min. Residual dyes were washed with PBS, and cells were resuspended in PBS for analysis of fluorescence by flow cytometer.

### Cell membrane depolarization

To test the effect of RPMs on cell membrane depolarization of *C. auris*, 1 × 10^6^ cells/mL were incubated with 200 µg/mL of FK20 for 45 min at 37°C with continuous shaking at 200 rpm. Treated and untreated cells were washed with PBS and resuspended with 5 µM bis-(1,3-dibutylbarbituric acid) pentamethine oxonol (Santa Cruz) to detect intracellular ROS by staining for 20 min. Cells were resuspended in PBS for analysis of fluorescence by flow cytometer.

### Statistics and reproducibility

All statistical analyses were performed using GraphPad Prism version 8.0.2. No statistical methods were used to pre-determine sample size. Sample sizes were based on previous publications that achieved statistically significant results (*P* < 0.05). Unless otherwise noted, all statistical analyses were performed with at least three biologically independent samples. All images are representative of a minimum of three biologically independent samples that represent a minimum of three independent experiments unless otherwise noted. For comparisons between two groups, two-tailed unpaired *t*-tests were performed.

## SUPPLEMENTAL MATERIAL

Supplemental material mbio.00400-26-s0001.pdf Supplemental figures, movie legend, and table.

Movie S1 mbio.00400-26-s0002.avi FK20 RPM penetrates *C. auris*.

ASM does not own the copyrights to Supplemental Material that may be linked to, or accessed through, an article. The authors have granted ASM a non-exclusive, world-wide license to publish the Supplemental Material files. Please contact the corresponding author directly for reuse.
