# Supplementary material for: Harnessing random peptide mixtures to combat multidrug-resistant fungal infections
Source: mBio. 2026 Apr 14;17(5):e00400-26. doi: 10.1128/mbio.00400-26 (PMC13170328; doi:10.1128/mbio.00400-26)
Supplement: Supplemental material — Supplemental figures, movie legend, and table. [file mbio.00400-26-s0001.pdf]

<u>Supplementary Data</u>

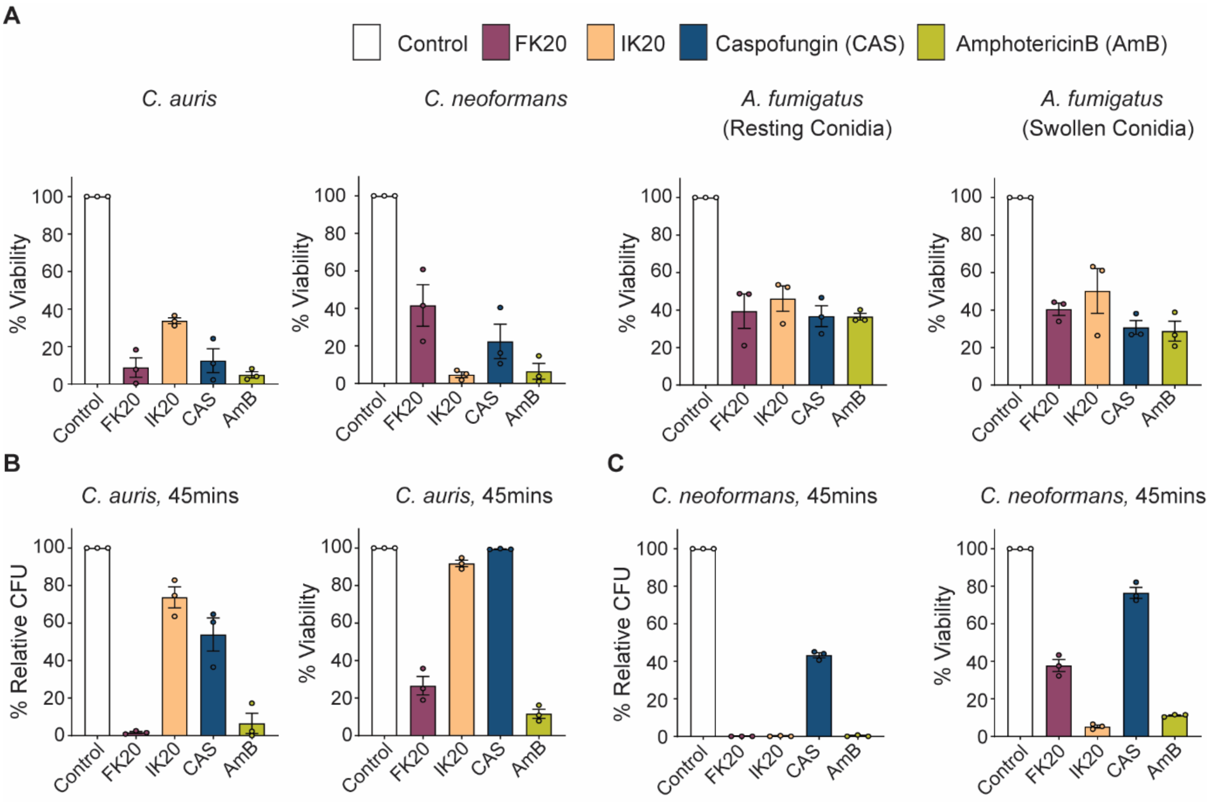

Figure S1: FK20 potency varies across major fungal pathogens of clinical relevance. A. *C. auris* and *C. neoformans* yeast cells, as well as *A. fumigatus* conidia (resting and swollen), were incubated with 200µg/ml FK20 or IK20 RPMs, 2µg/ml Caspofungin, 2µg/ml Amphotericin B, or PBS as a control for 5h at 37°C, followed by PI staining to determine cell viability **B-C**. *C. auris* and *C. neoformans* cells were treated as stated above for 45 minutes at 37°C. Fungal viability after treatment is presented for *C. auris* as percentage relative CFUs (B, left panel), % viability after PI staining (B, right panel) and for *C. neoformans* as percentage relative CFUs (C, left panel), % viability after PI staining (C, right panel). Data represents the mean ± SEM of three biologically independent experiments. Statistical significance was determined using student's t-test, comparing each treatment to the untreated control (set to 100% for each biological replicate). Significance levels are indicated as *p <0.05, **p < 0.01, ***p<0.001 and ****p < 0.0001; values without symbols are not significantly different from the control.

Supplementary Movie 1. FK20 RPM Penetrates C. auris. $5 \cdot 10^7$ CFU/mL of *C. auris* cells were incubated with 200 µg/mL fluorescein-labeled FK20 RPM (green) for 45 minutes at 37ºC in PBS. Samples were co-stained with Calcofluor White stain (blue). Z-stack movie prepared using Z images taken by a confocal microscope at a magnification of 40×. The video was edited using ImageJ. Scale bar, 10 µm.

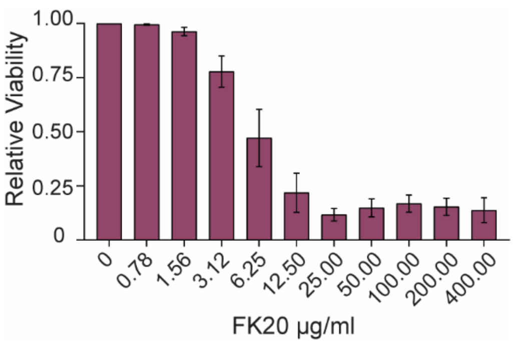

Figure S2: Membrane damage quantification by ghost dye staining. *C. auris* cells were incubated with FK20 RPM at concentration ranging from 0 – 400 µg/mL for 45 minutes at 37°C with continuous shaking. Membrane damage was evaluated by staining *C. auris* cells with Ghost dye for 20 minutes. Cell viability was determined by measuring Ghost dye fluorescence using a flow cytometer. The viability of *C. auris* was calculated as the percentage of cells that remained unstained by Ghost dye relative to the untreated control. The data are derived from three biologically independent experiments conducted in triplicates.

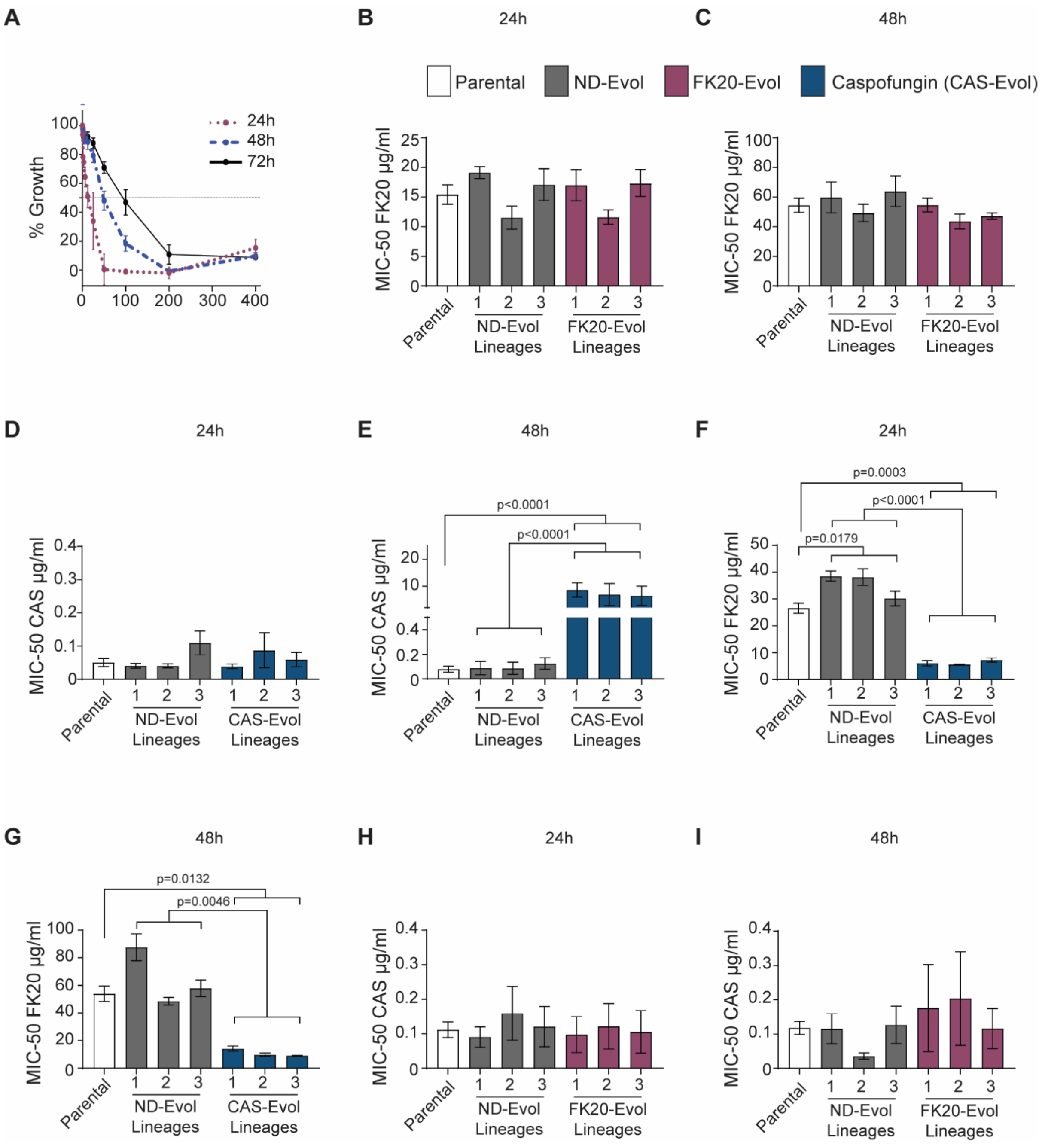

Figure S3. MIC determination and experimental evolution of *C. auris* strains at multiple time points.

(A) Initial MIC determination for FK20 against parental *C. auris* using broth microdilution assay. Growth curves were measured across FK20 concentrations (0–400 µg/mL) after 24, 48 and 72 hours. **(B–I)** MIC-50 values for parental, non-drug-evolved (ND-evol), and drug-evolved lineages under FK20 or caspofungin treatment at 24 hours **(B, D, F, H)** and 48 hours **(C, E, G, I)** post-exposure. (**B–C)**: FK20 MIC-50 for parental, ND-evol, and FK20-evolved lineages. **D–E**: Caspofungin MIC-50 for parental, ND-evol, and caspofungin-evolved lineages. (**F–G)**: FK20 MIC-50 for parental, ND-evol, and caspofungin-evolved lineages (collateral sensitivity). (**H–I)**: Caspofungin MIC-50 for parental, ND-evol, and FK20-evolved lineages (collateral sensitivity). Each data point represents the mean ± S.E.M. for the parental strains and the three independent lineages of evolved strains across three biological replicates. Statistical comparisons were performed using one-way ANOVA with Tukey's multiple comparisons test. Significance levels are indicated as *p<0.05, **p<0.01, ***p<0.001 and ****p<0.0001; values without symbols are not significantly different from the control.

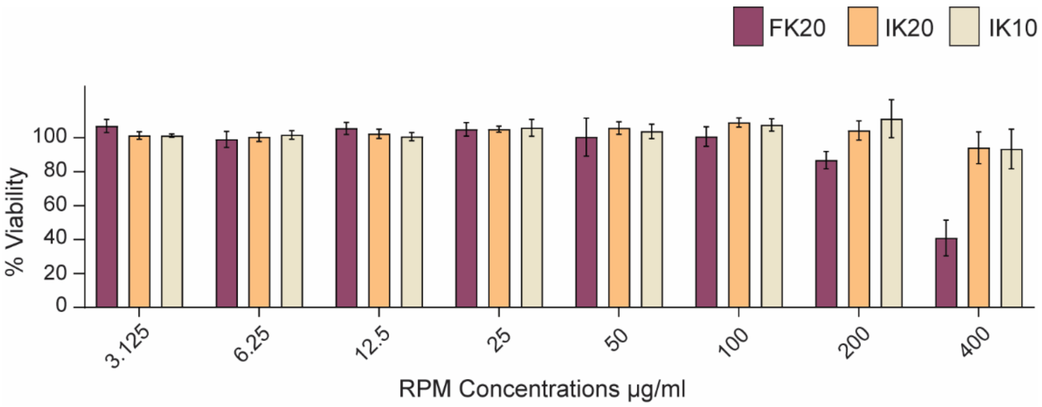

Figure S4: RPMs exhibit no cytotoxicity in cultured cells. RAW 264.7 murine macrophage cells were incubated with FK20, IK20, or IK10 for 24 hours at 37°C in a 5% CO_2 environment. Cell viability was assessed using the MTT assay, with macrophage viability expressed relative to a control group with no treatment. The data represents the mean of three biologically independent experiments, each performed in triplicate.

Test	UPW	IK10	FK20	Reference values
**AST (IU/L)**	341.5 ± 246.5	187 ± 81	275 ± 90	57-329
**ALT (IU/L)**	38 ± 20	28 ± 3	32 ± 6	22-133
**ALP (IU/L)**	141.5 ± 13.5	110.5 ± 12.5	94 ± 12	16-200
**GGT (IU/L)**	< 3	< 3	< 3	< 3
**Triglyceride (mg/dL)**	70 ± 37	54.5 ± 20.5	61 ± 10	16-164
**Cholesterol (mg/dL)**	84.15 ± 5.55	97.45 ± 3.75	85.5 ± 4	34-173
**Total Bilirubin (mg/dL)**	0.159	< 0.15	< 0.15	0.1-0.9
**Glucose (mg/dL)**	395 ± 178	219.5 ± 49.5	328 ± 34	60-133
**Albumin (g/dL)**	3.75 ± 0.55	3.8 ± 0.49	3.7 ± 0.1	2.6-5.4
**Total protein (g/dL)**	4.895 ± 0.095	4.71 ± 0.44	4.6 ± 0.1	4.6-7.3
**UREA (mg/dL)**	31.4 ± 0.9	35.9 ± 0.5	32 ± 3	2-71
**Creatinine (mg/dL)**	0.315 ± 0.015	0.32 ± 0.03	0.33 ± 0.03	0.1-1.8
**Phosphate (mg/dL)**	10.85 ± 3.85	8.875 ± 0.435	9.796 ± 0.6	5.3-11.3
**Ca (mg/dL)**	9 ± 0.9	9.25 ± 0.55	8.73 ± 0.33	6.8-11.9
**Na (mmol/l)**	145	149.5 ± 0.5	142 ± 1.2	145–155
**Cl (mmol/l)**	104.85 ± 1.25	110.55 ± 0.15	103 ± 1.36	105–115
**K (mmol/l)**	6.885 ± 0.98	6.385 ± 0.135	6.5 ± 0.46	6.5-9.7

Table S1: Mice blood chemistry. A control group of Mice were administered ultra-pure water, FK20 or IK10 RPMs intramuscularly daily for four days (25 µg/gr body weight). Blood chemistry was analyzed on day four. n=5 per group.

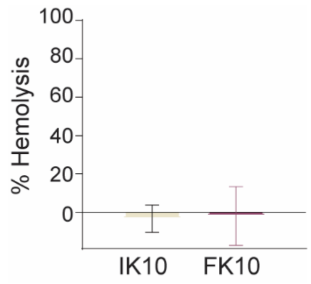

Figure S5: RPMs are not hemolytic. A control group of Mice were administered ultra-pure water, FK20 or IK10 RPMs intramuscularly daily for four days (25 µg/gr body weight). Hemolysis was analyzed on day four by direct spectrophotometric measurement at 540 nm. n=3 per groups.

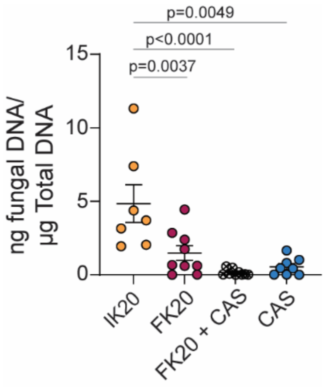

Figure S6: Quantification of fungal burden by qPCR in the murine systemic candidiasis model. Fungal burden was assessed by quantitative PCR (qPCR) on DNA extracted from kidney tissues of *C. auris* infected mice following treatment with IK20 (15mg/kg), FK20(15mg/kg/), Caspofungin (3mg/kg), or a combination of FK20 and Caspofungin (15mg/kg and 3mg/kg respectively). Fungal load was quantified on day 4 post-infection as ng of fungal DNA per µg of total DNA. Statistical analysis was performed using one-way ANOVA, and p-values for significant comparisons are indicated on the graph, comparisons without p-values are not statistically significant.
